# Patterns of genetic structure and adaptive positive selection in the Lithuanian population from high-density SNP data

**DOI:** 10.1038/s41598-019-45746-3

**Published:** 2019-06-24

**Authors:** A. Urnikyte, A. Flores-Bello, M. Mondal, A. Molyte, D. Comas, F. Calafell, E. Bosch, V. Kučinskas

**Affiliations:** 1 Department of Human and Medical Genetics, Biomedical Science Institute, Faculty of Medicine, Vilnius University, Santariskiu Street 2, LT-08661 Vilnius, Lithuania; 2 Institut de Biologia Evolutiva (UPF-CSIC), Departament de Ciències Experimentals i de la Salut, Universitat Pompeu Fabra, Parc de Recerca Biomèdica de Barcelona, Doctor Aiguader 88, 08003 Barcelona, Catalonia Spain; 3 Institute of Genomics, University of Tartu, Riia 23b, Tartu, 51010 Tartu Estonia

**Keywords:** Population genetics, Population genetics, Population genetics, Population genetics

## Abstract

The analysis of geographically specific regions and the characterization of fine-scale patterns of genetic diversity may facilitate a much better understanding of the microevolutionary processes affecting local human populations. Here we generated genome-wide high-density SNP genotype data in 425 individuals from six geographical regions in Lithuania and combined our dataset with available ancient and modern data to explore genetic population structure, ancestry components and signatures of natural positive selection in the Lithuanian population. Our results show that Lithuanians are a homogenous population, genetically differentiated from neighbouring populations but within the general expected European context. Moreover, we not only confirm that Lithuanians preserve one of the highest proportions of western, Scandinavian and eastern hunter-gather ancestry components found in European populations but also that of an steppe Early to Middle Bronze Age pastoralists, which together configure the genetic distinctiveness of the Lithuanian population. Finally, among the top signatures of positive selection detected in Lithuanians, we identified several candidate genes related with diet (*PNLIP*, *PPARD*), pigmentation (*SLC*2*4A5*, *TYRP1* and *PPARD*) and the immune response (*BRD2*, *HLA-DOA*, *IL26* and *IL22*).

## Introduction

Lithuania is a north-eastern European country where the most archaic Indo-European language is spoken^1^. The current Lithuanian population resulted from a complex amalgam between the former Baltic tribes each with potentially different contributions from Finno-Ugric and Slavic sources^2^. Moreover, since the Neolithic, the native population of what would become Lithuania has not been substituted by other peoples. Thus, the origin of the contemporary Lithuanian population may be traced back to the Neolithic settlers, with little admixture hereafter^3^.

The first settlement in the contemporary Lithuanian territory was founded in the late Palaeolithic in west Lithuania along the Baltic sea after the last glaciation around 11,000 years before present^4,5^. These people were highly related to hunter-gatherers (HG) from western Europe^4^. Notably, according to Lazaridis *et al*. (2014)^6^ modern eastern Baltic populations carry the largest proportion of western hunter-gatherer (WHG) ancestry of all Europeans. Moreover, recent results from Mittnik *et al*. (2018)^7^ point to continued gene flow between groups in the southeast Baltic region, who were more closely related to WHG, and the northern or eastern groups, more closely related to the eastern hunter-gatherers (EHG)^6,7^. Archaeological findings suggest that Finno-Ugrians had a minor contribution on the Lithuanian population^5^. However, Y-chromosome biallelic markers showed similarities between both Lithuanians and Latvians and the Finno-Ugric Estonians and Mari^8^. On the other hand, while mtDNA diversity also show this connection of Lithuanians to Finno-Ugric speakers, it also revealed similarities with Slavic (Indo-European) speaking populations^2^. Finally, analysis of molecular variance using the mtDNA HV1 region and Y STR haplotypes showed that Lithuania is a homogeneous population^2^.

The apparently long isolation of the Balts may have contributed to the preservation of an ancient social structure and of an archaic language^9^. In addition, the establishment of the Baltic tribes lead to the development of the current Lithuanian dialects, resulting in the actual regional linguistic differentiation. Six dialects can be currently distinguished in Lithuania: three groups from Aukstaitija (west, south and east) and three groups from Zemaitija (north, west and south).

The characterization of the adaptive history of local populations is of great interest because it provides knowledge about the genes that have been targeted by positive natural selection at a regional geographical scale. Besides their biological role in our past survival, these genes could be involved in rare, severe Mendelian diseases and contribute to current differences in resistance or susceptibility to disease^10^. Indeed, several genetic variants that were adaptive in the past have been suggested to currently associate to particular common immune response and metabolic disease phenotypes due to recent environmental and dietary shifts^11,12^. During the last years, the availability of high-throughput genotyping platforms and next generation sequencing techniques together with the increasing number of genotype-phenotype association studies and the development of new statistical and computational methods in the field of evolutionary genomics allow not only inferring putative selective events but also deciphering their potential phenotypic adaptive targets when analysing genomic patterns of variation from contrasting environmental conditions. To date many population genetics studies have been performed on large reference populations, but the need to analyse local patterns of population structure and adaptation remains. Without doubt, the analysis of geographically specific regions and the characterization of fine-scale patterns of genetic diversity may facilitate a much better understanding of the microevolutionary processes affecting local human populations.

To the best of our knowledge, there are no previous studies describing fine-scale genetic structure and recent positive selection in the Lithuanian population. Thus, in this study, we aimed to analyse local patterns of population structure and signatures of adaptive positive selection from genome-wide high–density SNP genotyping data we generated with the Illumina HumanOmmiExpress-12v1.1 and Infinium OmniExpress-24 arrays in 425 individuals from six regions in Lithuania. Furthermore, by combining our dataset with ancient data publicly available we aimed to explore the prehistorical ancestry components present in the Lithuania population. Notably, our results display Lithuanians as a homogenous population presenting one of the highest proportions of distinct pre-Neolithic HG (i.e. western, eastern and Scandinavian) and Early to Middle Bronze Age steppe pastoralist ancestry components found across contemporary Europeans. Moreover, among the top signatures of positive selection detected in Lithuanians we identified several candidate genes related to diet (*PNLIP*, *PPARD*), pigmentation (*SLC24A5*, *TYRP1*, *PPARD*) and the immune response (*BRD2*, *HLA-DOA*, *IL26* and *IL22*).

## Results

### Genetic homogeneity across Lithuanian ethnolinguistic groups

**Figure 1:**
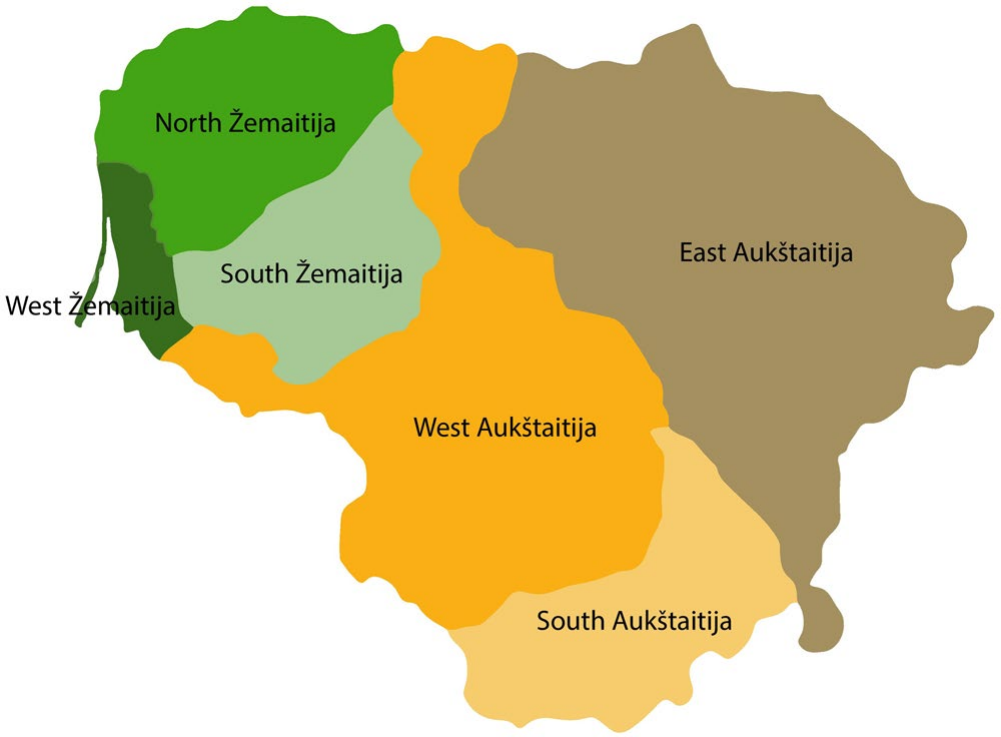
Map of Lithuanian ethnolinguistic groups. Six regions based on dialect are distinguished in Lithuania: three groups from Aukstaitija (west, south and east) and three groups from Zemaitija (north, west and south).

**Figure 2:**
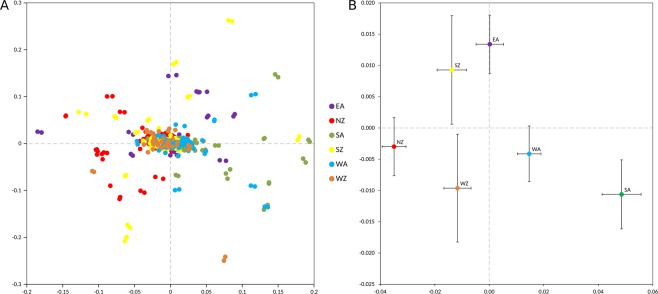
Principal component analysis (PCA) of the six ethnolinguistic groups of the Lithuanian population. (**A**) Principal components 1 and 2 (after removing outliers) are shown. NZ, North Zemaitija; SZ, South Zemaitija; WZ, West Zemaitija; EA, East Aukstaitija; SA, South Aukstaitija; WA, West Aukstaitija. (**B**) Centroids of the PC scores by ethnolinguistic groups. Bars represent standard errors.

We compiled and genotyped DNA samples from 425 Lithuanian individuals belonging to the six ethnolinguistic groups nowadays present in the Aukstaitija (western (n = 79), southern (n = 67), and eastern (n = 79)) and the Zemaitija (northern (n = 79), western (n = 43), and southern (n = 78)) regions of Lithuania (Fig. 1). Genetic inbreeding and similarity between all possible pairs of individuals in the Lithuanian population was investigated using the genome-wide SNP data generated through the kinship^13^ and the inbreeding coefficients^14^, which were estimated at 0.00075 and 0.0022, respectively. Notably, four individuals had F values higher than expected for second cousin mating offspring (0.0156), and 19 pairs of individuals (including 1 duplicate) were inferred to present kinship coefficients higher than expected for 2nd degree relatives (0.0084). Moreover, a first exploratory principal component analysis (PCA) revealed 8 outliers (Fig. S1), which were subsequently removed from further analyses together with the aforementioned related and inbred individuals detected (Table S1, Fig. S2). Next, the genetic relationships of the six Lithuanian ethnolinguistic groups were explored by performing a second PCA using the 232,752 genome-wide pruned SNPs successfully genotyped in the remaining 399 Lithuanian samples. At that scale, the first two principal components (PC) explaining 0.81% of the variance showed that the six ethnolinguistic groups apparently formed a single cluster (Fig. 2A). However, when the average PC scores were computed for each ethnolinguistic group (Fig. 2B), a clear separation emerged. Both PC1 (F = 30.108, P = 7 × 10^−26^) and PC2 (F = 2.586, P = 0.026) were statistically significantly different among ethnolinguistic groups. If the main regions were compared to each other (Aukstaitija vs. Zemaitija), only PC1 scores were significantly different (F = 83.443, P = 3 × 10^−18^). Still, genetic differentiation assessed through calculation of pairwise *F*_*ST*_ values revealed that the fraction of the genetic variance due to differences among ethnolinguistic groups ranged from 0.19% to 0.39% (Table 1). Thus, even if statistically significant, the genetic differentiation among the Lithuanian ethnolinguistic groups is quantitatively small.

**Table 1:** Weir and Cockerham *F*_*ST*_ estimates between pairs of ethnolinguistic groups in Lithuania.

Population	NZ	SZ	WZ	EA	WA	SA
NZ	0					
SZ	0.001886	0				
WZ	0.003293	0.00333	0			
EA	0.002236	0.002485	0.003764	0		
WA	0.002006	0.00217	0.003641	0.001986	0	
SA	0.002738	0.002586	0.003884	0.002765	0.002201	0

NZ, North Zemaitija; SZ, South Zemaitija; WZ, West Zemaitija; EA, East Aukstaitija; SA, South Aukstaitija; WA, West Aukstaitija.

### Population history of Lithuania

**Figure 3:**
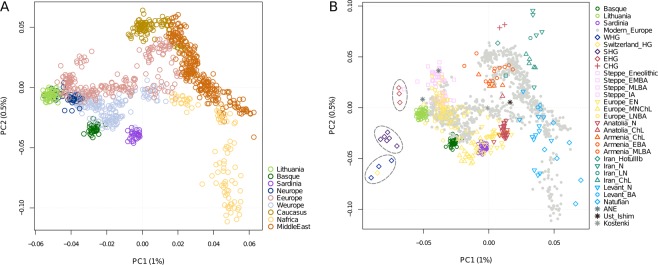
Lithuania within Western Eurasia. (**A**) PCA based on present-day samples from West Eurasia and North Africa. **(B**) PCA based on present-day samples, with ancient samples projected. Present-day samples are shown as grey points, with the exception of Lithuanians (light green), Basques (dark green) and Sardinians (purple). E, Early; M, Middle; L, Late; HG, Hunter-gatherer; N, Neolithic; ChL, Chalcolithic; BA, Bronze Age; IA, Iron Age. Western HG, Scandinavian HG and Eastern HG are circled.

To obtain a broader continental and historical context for the genetic diversity of the contemporary Lithuanian population, we next combined the autosomal data generated here with that of the Human Origins dataset from Lazaridis *et al*. (2016)^6^ including a wide range of modern and ancient samples from west Eurasia and north Africa. Principal component analysis (PCA) of all modern samples displayed a tight clustering of the Lithuanian samples partly overlapping the north European group and in close proximity towards one of the extremes of the eastern and western European clusters (Fig. 3A). Interestingly, when adding ancient samples from different historical periods, Lithuanians positioned as the closest present-day European population to three different pre-Neolithic HG groups (the western, the Scandinavian and the eastern HG) and showed high proximity to the Steppe and Late Neolithic Bronze Age (LNBA) European samples (Fig. 3B).

ADMIXTURE analysis at *K* = 4 (the lowest cross-validation error) revealed three ancestry components on the Lithuanian population (Fig. S3): one major component reflecting the HG ancestry preserved in Lithuanians (in green), and two additional lower genetic components found maximized in the European Neolithic/Anatolian Neolithic (in orange) and in the Iran Neolithic (in blue) farmers, respectively. Notably, this latter component is also highly present in the Early to Middle Bronze Age (EMBA) Steppe pastoralists, from which it arrived to the Baltic and the rest of Europe. Furthermore, when compared to their contemporary neighbouring populations in western and northern Europe (West Europe and North Europe groups in Fig. S3), Lithuanians seem to lack the Natufan/Levant Neolithic ancestry component (in purple) present in them; on the contrary, Lithuanians carry higher proportions of HG ancestry.

**Figure 4:**
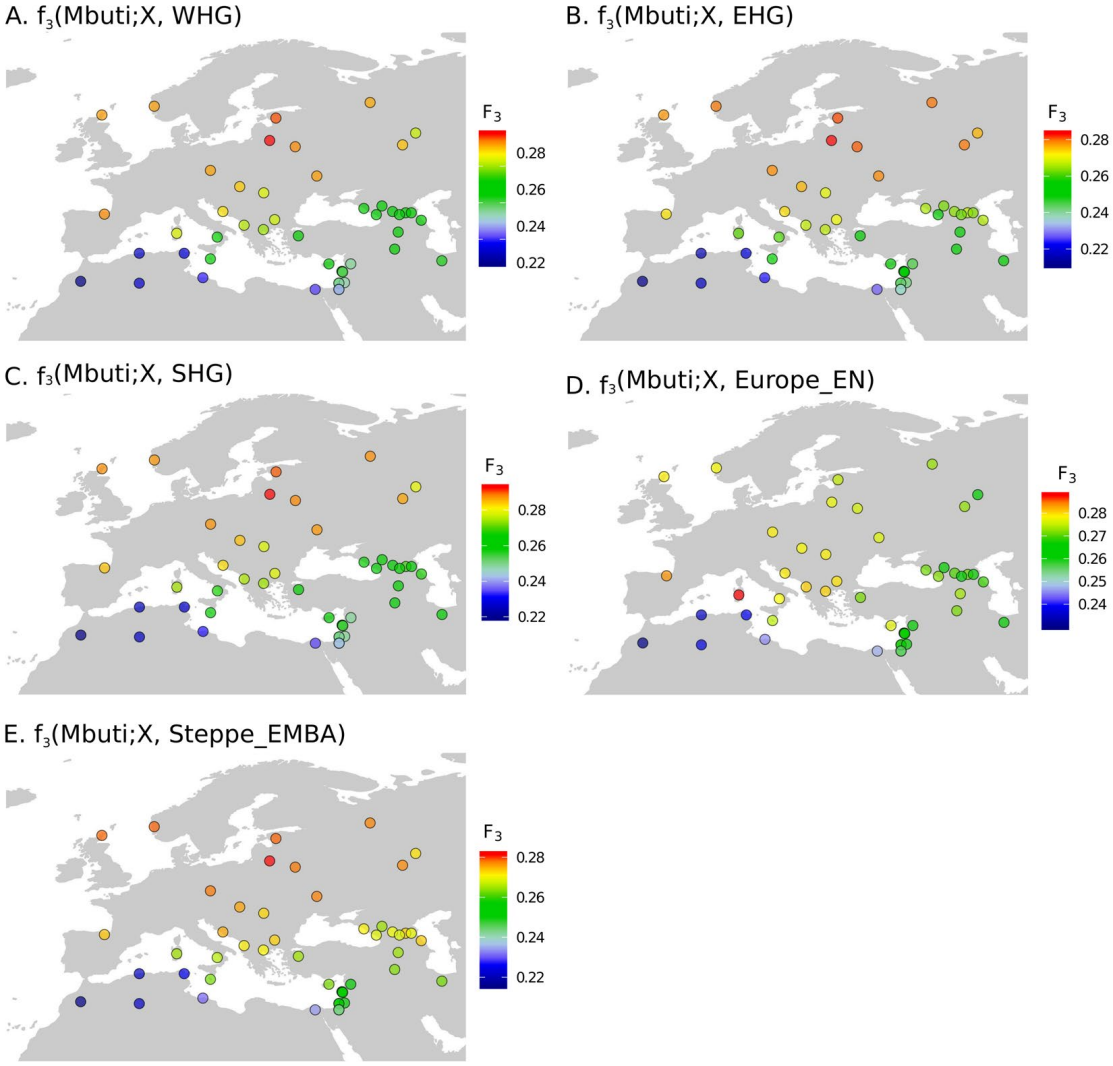
Geographic distribution of outgroup f_3_-statistics. Outgroup f_3_ values for the top ancient ancestries detected in the Lithuanian population were plotted across contemporary populations in western Eurasia: (**A**) f_3_(Mbuti; X, WHG). (**B**) f_3_(Mbuti; X, EHG). (**C**) f_3_(Mbuti; X, SHG). (**D**) f_3_(Mbuti; X, Europe_EN). (**E**) f_3_(Mbuti; X, Steppe_EMBA), being X a modern population.

Next, we used outgroup f3 statistics^15^ to formally explore the presence and geographical distribution of the main ancestry components detected in Lithuania across western Eurasia. Among the contemporary populations of the Lazaridis *et al*. (2016)^6^ dataset, Lithuanians display the most extreme admixture f3 values when specifically testing the western HG ancestry in the form f3 (Mbuti; contemporary population, WHG), as well as the ancestry components of Scandinavian HG, the eastern HG, and the EMBA steppe pastoralists but not for the European Neolithic farmer component characterizing the genetically isolated Sardinians and Basques (Fig. 4). Similarly, no significant allele sharing was detected between Lithuanians and Anatolian Neolithic, Levant Neolithic or Caucasus HG, whereas Lithuanians also presented top f3 values for the posterior Late Neolithic Bronze Age (LNBA) European component (Fig. S4).

### Genetic adaptation in the Lithuanian population

**Figure 5:**
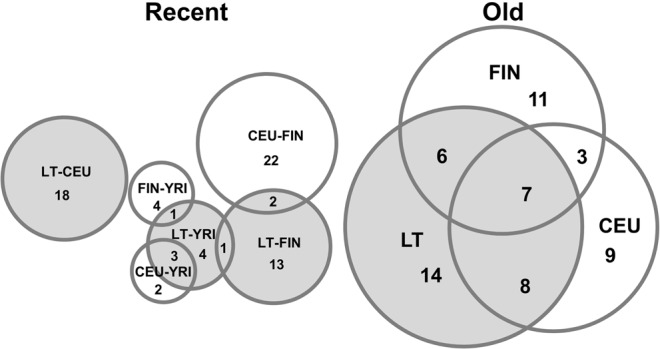
Shared signals of adaptation between Lithuanians and the CEU and FIN populations from the 1000 Genomes Project Phase3 dataset. Venn diagrams showing the overlap of candidate regions for recent and older events of positive selection as detected by the combined *F*_*ST*_ and XP-EHH approach and the Tajima’s D statistic, respectively.

Before analysing signatures of positive selection, we merged our original genotyping data in the Lithuanian population to an additional, but much denser, external SNP dataset, namely the 1000 Genomes Project Phase3 dataset^16^. PCA and admixture analysis confirmed the expected clustering of Lithuanians within the European context as well as the consistent genetic differentiation and grouping of all six Lithuanian ethnolinguistic groups when compared to CEU, FIN, GBR and TSI (Supplementary Note 1). To detect putative recent selective events, we next calculated *F*_*ST*_ and XP–EHH between all possible pairs of populations among LT, CEU, FIN and YRI, and focussed on those signals found in the Lithuanian population. For each population comparison, the genome-wide distribution of signals detected with *F*_*ST*_ and XP-EHH is shown in Figs S5 and S6, respectively. In each comparison, we considered as top candidates for recent selection those genomic regions presenting at least 2 SNPs over the top 0.1% XP-EHH empirical values and a minimum of 1 SNP with an *F*_*ST*_ rank score p-value < 0.01. Thus, we detected a total of 42 candidate regions with signatures of recent selection in the Lithuanian population (Table 2). Interestingly, most recent signals were found when comparing Lithuanians to either the CEU (18 candidate regions) or the FIN populations (16 signals). Moreover, out of the nine signals identified when comparing LT to YRI, three were also detected in the CEU-YRI comparison and an additional one in the FIN-YRI comparison and thus indicate shared signatures of selection when comparing the three European populations to YRI (Fig. 5). Among them, we detected a ~296 kb region in chromosome 15, comprising the *SLC24A5* gene, which is a known target of recent positive selection favouring light pigmentation in non-African populations^17^. Accordingly, the intergenic variant rs1834640, reported to be associated to skin pigmentation^18^, was found as one the six top combined XP-EHH and *F*_*ST*_ significant values in the CEU-YRI comparison, whereas in the LT-YRI comparison only rs12440301 (~2,500 bp apart from rs1834640) displayed such a pattern in the same region.

**Table 2:** Candidate regions under recent positive selection in the Lithuanian population as detected by *F*_*ST*_ and XP–EHH.

Genome coordinates	Genes	Population (SNPs^a^)
chr1:35484861–35635453	*ZMYM6, ZMYM1, SFPQ*	LT–YRI (3)
chr1:36549157–36562551	*TEKT2, COL8A2*	LT–YRI (3)
chr1:231866461–231908347	*TSNAX-DISC1*	LT–CEU (3)
chr1:245363423–247401645	*KIF26B*	LT–FIN (4)
chr2:56070352–56161538	*PNPT1,EFEMP1*	LT–CEU (4)
chr3:2347409–2355167	*CNTN4*	LT–FIN (5)
chr3:112511598–112816192	*LOC101929694, CD200R1L*	LT–FIN (5)
chr3:130096881–131445951	*COL6A5, COL6A6, CPNE4*	LT–FIN (2), LT-YRI (31)
chr4:2389513–23901275	*PPARGC1A, MIR573*	LT-CEU (4)
chr4:106552414–106594625	*ARHGEF38*	LT–YRI (3), FIN–YRI (3)
chr4:14879847–148916437	*ARHGAP10*	LT-YRI (2), CEU-YRI (10)
chr4:89629000–89669852	*HERC3,FAM13A*	LT–FIN (4)
chr4:181637915–181691738	*LINC00290*	LT–FIN (4)
chr5:10073956–10109791	*LOC285692, FAM173B*	LT–CEU (4)
chr5:133177588–133638034	*FSTL4, WSPAR, CDKL3*	LT–FIN (4)
chr5: 165752621–165758259	*LOC102546299, CTB-7E3.1*	LT–FIN (2)
chr6: 3205325–3207258	*LOC100507194, TUBB2B*	LT–FIN (2)
chr6:11710405–11717934	*TMEM170B, ADTRP*	LT–FIN (4)
chr6:27811815–28096280	*HIST1H2BN, HIST1H2AL, HIST1H1B, HIST1H4L, HIST1H3J, HIST1H2BO, OR2B2, OR2B2, OR2B6, ZNF165, ZSCAN12P1, ZSCAN16-AS1,GPX5*	LT–FIN (43)
chr6:32961621–32965942	*BRD2, HLA-DOA*	LT-CEU (3)
chr6:46596286–46834685	*CYP39A1, PLA2G7, MEP1A, ADGRF5*	LT–CEU (8)
chr7:21298242–21346861	*LINC01162, SP4*	LT-CEU (2)
chr7:30276161–30415102	*MTURN, ZNRF2, DKFZP586I1420, LINC01176*	LT–YRI (5)
chr7:112994204–113020044	*LINC00998, PPP1R3A*	LT–FIN (4)
chr7:129187430–129350170	*SMKR1, NRF1*	LT–CEU (11)
chr9:12352971–12537279	*PTPRD-AS2,TYRP1*	LT–CEU (29)
chr9:129633328–129646775	*ZBTB34*	LT–CEU (2)
chr10:116362835–116377962	*ABLIM1*	LT–FIN (4)
chr11:41897826–42307166	*LINC01499, LOC100507205, HNRNPKP3*	LT–YRI (10)
chr11:60047410–60107105	*MS4A4A*	LT–CEU (3)
chr11:129457972–129458962	*BARX2, LINC01395*	LT–CEU (2)
chr11:129832270–130645506	*PRDM10,LINC00167,APLP2,C11orf44,LOC100507431*	LT–YRI (10), CEU–YRI (7)
chr12:68640583–68654780	*IL26, IL22*	LT–CEU (6)
chr12:103326468–103394540	*PAH, ASCL1*	LT–CEU (5)
chr13:101364651–101375663	*NALCN-AS1*	LT-CEU (2)
chr15:48387088–48686175	*LINC01491, SLC24A5, SLC12A1, DUT, FBN1*	LT–YRI (8), CEU–YRI (16)
chr15:51639821–51673630	*GLDN*	LT–CEU (9)
chr15:58579956–58588116	*AQP9, LIPC*	LT–CEU (4)
chr16:66962411–67028008	*RRAD, FAM96B, CES2, CES3, CES4A*	LT–FIN (6)
chr17:4722606–4943176	*PLD2, MINK1, CHRNE, GP1BA, SLC25A1, RNF167, ENO3, SPAG7, CAMTA2, KIF1C, SLC52A1, ZFP3*	LT–FIN (15), CEU-FIN (11)
chr20:19702049–19706617	*SLC24A3, RIN2*	LT–FIN (6), CEU–FIN (13)
chr21:21389233–21568418	*MIR548XHG, LINC00320*	LT–CEU (8)

^a^Number of significant SNPs that were located at the extreme 0.1% of the empirical distribution for the XP–EHH and at least one SNP in the region had *F*_*ST*_ value < 0.01.

Another of the strongest signals detected in the LT-YRI comparison was found in a ~225 kb region in chromosome 3, which comprises the *COL6A5* and *COL6A6* genes coding for the collagen type VI alpha 5 and alpha 6 chains, respectively. Interestingly, a non-synonymous variant in *COL6A5* (rs12488457: 3838A > C, Thr1280Pro) with a CADD value of 23.2 was found among the top XP-EHH and *F*_*ST*_ outliers along the region. Moreover, the SNP is reported as an eQTL for *COL6A5* in testis (P = 3.0 × 10^−17^) and for *COL6A6* in heart (P = 4.1 × 10^−9^) with the derived C allele implying lower expression in both cases according the Genotype-Tissue Expression (GTEx) portal (https://gtexportal.org/home/; accessed on 28/02/19). Notably, even if the signal was only significant in the LT-YRI comparison, all European populations present high frequencies (above 0.748) for the derived C allele at rs12488457, which is found at very low frequencies in YRI (~0.013). Finally, an additional signal in the LT-YRI comparison mapping on chromosome 1 includes two extreme XP-EHH and *F*_*ST*_ outliers (rs274750 and rs274752) at the 3′UTR of the *COL8A2* gene, which codes for the collagen type VIII alpha 2 chain. Again, the signal was only significant in the LT-YRI comparison but the derived alleles at rs274750 and at rs274752, C and A respectively, are nearly fixed in the three European populations and found at intermediate frequencies in YRI. Interestingly, polymorphisms in *COL6A5* have been associated with body mass index^19^ and dermal phenotypes, such as eczema^20^, while mutations at *COL8A2* have been linked to corneal endothelial dystrophies^21^.

As for the LT-CEU comparison, one of the strongest signals detected in Lithuanians was found at a ~184 kb region in chromosome 9 that comprises the *TYRP1* gene, which codes for a melanosomal enzyme that participates in the melanin biosynthetic pathway. However, no obvious functional variant was identified among the top outlier XP-EHH and *F*_*ST*_ values in that region. In the same population comparison we also identified two signals probably related to immunity: one in chromosome 12 comprising the *IL26* and *IL22* interleukin genes, and the other in chromosome 6 containing the *BRD2* and *HLA* genes.

A total of 35 candidate regions for older signatures of selection were identified in the Lithuania population when using the Tajima’s D statistic (Table 3, Fig. S7). Notably, 21 of these candidate regions were also detected in CEU (8), in FIN (6) or in both CEU and FIN (7) pointing to a high proportion of common old targets of selection among Europeans (Fig. 5). That is the case of the *SLC24A5* region related to light pigmentation which, besides being identified as target of recent selection in the LT-YRI and CEU-YRI comparisons, emerged also as an outlier for old signatures in all three European populations. Interestingly, at least two of the candidate regions detected in Lithuanians for old signatures of selection may be related to dietary and environmental pressures: one in chromosome 6 containing the *PPARD* gene and another in chromosome 10 comprising the *PNLIP* and *PNLIPRP3* genes. Whereas *PNLIP* codes a pancreatic lipase required for the efficient digestion of dietary fats^22^, *PPARD* stimulates beta-oxidation not only in muscle and adipose tissue but also in the liver and other tissues and has been suggested to be involved in metabolic adaptation to environmental changes^23^. However, in none of these two cases any obvious functional variant specifically linked to the detected signatures was identified among the SNPs genotyped.

**Table 3:** Candidate regions under positive selection in the Lithuanian population as detected by the Tajima’s D statistic.

Genome coordinates	Windows	P-value	Genes	Shared signal
chr1:35818960–35948959	4	0.0008	*KIAA0319L;ZMYM4*	
chr1:49988960–50728959	6	0.0003	*AGBL4;ELAVL4*	
chr1:188788960–188968959	9	0.0003	*LINC01037;BRINP3*	CEU
chr2:21728675–21888674	7	0.0004	*TDRD15;LINC01822*	FIN
chr2:179468675–179648674	9	0.0003	*TTN*	CEU
chr3:50343412–51893411	11	0.0006	*C3orf18;CACNA2D2;CISH;CYB561D2;DCAF1;DOCK3;GRM2;HEMK1;HYAL2;IQCF3;IQCF6;MANF;MAPKAPK3;NPRL2;RAD54L2;RASSF1;RBM15B;TEX264;TMEM115;TUSC2;ZMYND10*	CEU
chr3:128763412–128903411	5	0.0006	*CNBP;GP9;ISY1;ISY1-RAB43;RAB43*	CEU, FIN
chr3:143543412–143683411	5	0.0001	*SLC9A9*	CEU, FIN
chr4:171930684–172360683	4	0.0002	*LINC02431;MIR6082*	
chr4:176190684–176400683	11	0.0005	*ADAM29;GPM6A*	FIN
chr5:50531164–50691163	6	0.0003	*ISL1*	
chr5:126311164–126441163	4	0.0005	*C5orf63*	
chr6:35265879–35395878	4	0.0005	*DEF6;PPARD*	FIN
chr6:97855879–98005878	6	0.0007	*MIR548H3*	
chr7:30280729–30470728	8	0.0006	*NOD1;ZNRF2*	CEU
chr7:151730729–151870728	5	0.0002	*GALNT11;KMT2C*	CEU, FIN
chr8:48621077–48801076	9	0.0007	*CEBPD;PRKDC;SPIDR*	
chr8:93731077–93901076	8	0.0002	*TRIQK*	CEU
chr9:38474202–38614201	5	0.0001	*ANKRD18A*	FIN
chr9:125434202–125574201	5	0.0006	*OR1K1;OR1L3;OR1L4;OR1L6;OR5C1*	
chr10:66065709–66215708	6	0.0002	*REEP3;ANXA2P3*	FIN
chr10:118195709–118325708	4	0.0008	*PNLIP;PNLIPRP3*	
chr11:71554229–71734228	9	0.0004	*DEFB131B;IL18BP;NUMA1;RNF121*	
chr12:1296235–1426234	4	0.0009	*ERC1*	CEU, FIN
chr12:15736235–15886234	6	0.0003	*EPS8;PTPRO*	CEU, FIN
chr13:34108565–34278564	8	0.0005	*STARD13*	
chr14:64046743–64236742	10	0.0005	*SGPP1;WDR89*	
chr15:48357093–48477092	3	0.0005	*MYEF2;SLC24A5*	CEU, FIN
chr15:69617093–69737092	3	0.0002	*KIF23;PAQR5*	CEU
chr16:67321264–67501263	7	0.0005	*ATP6V0D1;HSD11B2;KCTD19;LRRC36;PLEKHG4;TPPP3;ZDHHC1*	CEU
chr17:29252345–29392344	5	0.0002	*ADAP2;LOC107984974;RNF135*	CEU, FIN
chr18:30389383–30559382	8	0.0001	*CCDC178*	CEU
chr19:50580913–50700912	3	0.0005	*IZUMO2*	
chr20:58399095–58539094	5	0.0006	*CDH26;FAM217B;PHACTR3;PPP1R3D;SYCP2*	FIN
chr21:44859932–44989931	3	0.0005	*HSF2BP*	

## Discussion

Geographically specific microevolutionary processes can be inferred when exploring local patterns of population structure and adaptation within the global and historical genetic context established from large general population and ancient publicly available reference panels. In this study we aimed to do that by analysing genome-wide high–density SNP genotyping new data generated in six ethnolinguistic groups from Lithuania.

At regional level, we found small genetic distances and a rather homogeneous genetic landscape across the six ethnolinguistic groups present in Lithuania. Previous analyses showed only statistically significant differences in the allele frequencies of the P1 and LWb blood groups when comparing south Aukstaitija with the remaining Lithuaninan ethno-linguistic regions as well as on the Alu TPA25 allele distribution between north Zemaitija and south Aukstaitija^24^. Similarly, according to Kasperaviciūtė *et al*. (2004)^2^, when considering Y-chromosome and mtDNA uniparental markers, Lithuania is a genetically homogeneous population. However, the sample size (180–196 Lithuanian samples) used in that study could be too small to identify minor differences between regions of Lithuania. Here, by increasing sample size to 399 individuals and covering the whole genome, we had statistical power to detect weak signals of genetic structure. However, even if we detected statistically significant genetic differentiation among the six Lithuanian ethnolinguistic groups, genetic distances among these groups were found to be rather small.

Archaeological findings locate the first settlement in the contemporary Lithuanian territory in the late Palaeolithic along the Baltic sea in west Lithuania (Zemaitija region), after the last glaciation around 11,000 years before present, probably favoured by the environment and the presence of marine food resources^4,25^. In contrast, the middle Lithuanian land was unsuitable to live permanently due to the extreme cold climate before 11,000 years ago^5^. People in the different regions of Lithuania seem to have lived in relative isolation for a long time because of the inaccessible nature of the terrain, bounded on the landward side by vast forests and swamps^26,27^. Thus, if genetic differences between Lithuanians from different geographical regions existed, these disappeared in the contemporary population. Alternatively, the genetic composition of the Baltic peoples from which contemporary Lithuanians originated may have been relatively homogeneous.

When combining the new data we generated with external datasets, we confirmed that Lithuanians locate within the expected European context, even though they also present particular genetic distinctiveness when compared to neighbouring populations. In addition, the inclusion of ancient individuals from different periods across western Eurasia in the analysis allowed us to distinguish the genetic signature of three main prehistorical sources shaping the distinctiveness of present-day Lithuanians: pre-Neolithic HG groups, the Early to Middle Bronze Age Steppe pastoralists and Late Neolithic Bronze Age (LNBA) Europeans. Moreover, up to three HG populations can be inferred to contribute to the main genetic component identified the Lithuanians being the contribution of the WHG and the Scandinavian HG greater than that of the EHG. On the contrary, earlier European Neolithic movements from Levant/Anatolia known to contribute to genetically differentiated populations in Europe such as Sardinians or Basques are not especially predominant in Lithuania.

Partial genetic isolation of the Lithuanians is a possible explanation for the structure results observed. Until the late Middle Ages, the eastern Baltic region was one of the most isolated corners of Europe^27^. Moreover, after the fall of the Roman Empire in the 5th century, the eastern Baltic region was spared by the subsequent population movements of the Migration Period^26,28^, which allowed the most archaic of all the living speaking Indo-European languages^1^to survive. Thus, Lithuanians could retain their cultural identity.

After establishing the genetic homogeneity and distinctiveness of Lithuanians within the European context, we next investigated whether specific signals of positive selection could be identified using different statistical approaches for recent and old classical selective sweeps. The two strongest signals for recent selection in our analysis comprise two candidate genes coding for different collagen chains (*COL6A5* and *COL6A6*) as well as the *TYRP1* gene involved in pigmentation and were detected in the LT-YRI and LT-CEU comparisons, respectively. Interestingly, an additional collagen gene (*COL8A2*) and a well-known target of positive selection related skin pigmentation (*SLC24A5*) also emerged among the top signals of recent selection detected in the LT-YRI comparison. Similarly, the two candidate regions for recent selection detected in the LT-CEU comparison comprising only genes related to the immune response (*IL26*, *IL22* and *BRD2*, *HLA-DOA*) and two candidate regions for old signatures in LT with genes probably related to diet (*PNLIP*, *PNLIPRP3* and *PPARD*) may probably result from specific pathogen driven and local dietary selection pressures in the Lithuania population. Notably, a significant number of the candidate regions for positive selection detected in the Lithuania population were also identified in FIN and/or CEU in our analyses and thus point to common selection signals across Europeans. Some of these signals had already been described in previous genome-wide scans of positive selection in Europeans and include the aforementioned *SLC24A5* gene as well as other candidates not always completely characterized such as *LRRC36*^29,30^ and *PPARD*^31^.

Other forms of adaptation resulting from soft sweeps such as polygenic selection or selection from standing variation have not been explored here and may provide additional insights to the past selective pressures of Lithuanians. Moreover, in most candidate regions the putative adaptive variants targeted by selection remain elusive since our genotyping data does not cover all the genetic variants present in each genomic region. However, even when the real adaptive variants are genotyped, their predicted functional relevance and subsequent assignation as putative adaptive is not always clear because different in silico prediction methods often provide non-congruent results. Despite all these limitations, we have identified several plausible candidate regions for selection in the Lithuanian population as well as some potential adaptive alleles, which after their subsequent experimental validation in follow-up studies may allow inferring new cases of local adaptation in the future.

## Material and Methods

### Samples

The initial data set consisted of 425 samples from individuals who reported a minimum of three generations of Lithuanian nationality. The average age of the participants was 53 years. Lithuania is divided in two main regions (Aukstaitija and Zemaitija), which can be further subdivided ethnolinguistically into six dialectal groups. We sampled three groups from Aukstaitija (western (n = 79), southern (n = 67), and eastern (n = 79)) and three groups from Zemaitija (northern (n = 79), western (n = 43), and southern (n = 78)) (Fig. 1). All the study participants provided written informed consent in accordance with the Declaration of Helsinki.

Genomic DNA was obtained from whole blood using either a standard phenol-chloroform method of extraction or the automated DNA extraction platform TECAN Freedom EVO (TECAN Group Ltd., Männedorf, Switzerland). A NanoDropR ND-1000 spectrophotometer (NanoDrop Technologies Inc., USA) was used to assess DNA concentration and quality. This work is part of the LITGEN project, which was approved by the Vilnius Regional Research Ethics Committee 235 No. 158200-05-329-79, date: 3 May 2011.

### Genotyping

Genotyping was performed at the Department of Human and Medical Genetics, Biomedical Science Institute, Faculty of Medicine, Vilnius University, Lithuania with the Illumina HumanOmniExpress-12v1.1 (296 samples) and the Infinium OmniExpress-24 (129 samples) arrays (Illumina, San Diego, CA, USA), which include an overlap of 707,138 genome-wide SNPs. Genotyping data quality control was performed according to the standard manufacturer recommendations. Individuals and SNPs with >10% missing data and SNPs with minor allele frequency (MAF) <0.01 were excluded from the analysis. SNPs with deviations from Hardy–Weinberg equilibrium (P < 10^−4^) were eliminated from the study. After quality control 1 individual was excluded with more than 10% missing genotypes (MIND >0.1) and 532,836 autosomal SNPs remained out of 589,752.

### European context and ancient data

To obtain a broader geographical and historical context for the genetic diversity of the Lithuanian population, we merged our genotyped autosomal Lithuanian data with that of the Human Origins dataset from Lazaridis *et al*. (2016)^6^. Next, the whole Lithuanian dataset was randomly sampled to keep a sample size of 50 individuals and avoid any potential overrepresentation in subsequent analyses. After this procedure, we ended up with a total of 1,033 modern and 284 ancient samples, respectively, and 52,477 SNPs after LD pruning.

### Principal component analysis, admixture and outgroup f3-statistics

Principal component analysis (PCA) for the six Lithuanian ethnolinguistic groups was carried out with independent pruned SNPs using SmartPCA from EIGENSOFT (v7.2.1)^32^. SNPs in linkage disequilibrium were removed with the indep-pairwise option of PLINK (v1.07) using a window size of 50 SNPs, a step size of 5, and a *r*^*2*^ threshold of 0.5^33^. Genetic relationship and consanguinity were inferred through the kinship and the inbreeding coefficients, which were estimated with KING v.2.1^13^ and PLINK v1.07^33^, respectively. Moreover, negative F values were converted to zero as they probably represent sampling errors^34^. Outlier samples on the PCA plot, as well as individuals with inbreeding coefficients higher than that expected for second cousin mating offspring (F values ≥ 0.0156) and 2nd degree relatives (kinship coefficient >0.0084) were removed for all subsequent analyses (Table S1).

To visualize the closest ancient groups to our modern Lithuanians, we performed a PCA on the merged Lithuanian – Lazaridis dataset^6^ with the SmartPCA program (v. 13050) from EIGENSOFT package (v7.2.1)^32^. The analysis was first carried out on the present-day Eurasian samples, and subsequently we projected the ancient individuals in the analysis. PCA results were then plotted in R (v3.2.3).

Model-based individual ancestries were estimated with ADMIXTURE (v. 1.3.0)^35^. We used the unsupervised method implemented in the program including both ancient and present-day samples with *K* (number of distinct ancestral clusters) ranging from 2 to 12. The results of 10 iterations were combined and plotted by using PONG (v1.4.7)^36^.

Outgroup f3-statistics were computed using the qp3Pop program from ADMIXTOOLS package (v4.1)^15^. We considered Mbuti as outgroup in the analysis and calculated the shared drift between each putative ancient group and all the modern groups in the dataset in the form f3 (Mbuti; Ancient, Modern). We have focused the analyses on the three main European ancestral components: pre-Neolithic HG (western, eastern and Scandinavian), European Neolithic farmer and Bronze Age steppe components. These groups are represented in the analyses as in the reference^6^ WHG, EHG, SHG, Europe_EN, Steppe_EMBA, respectively.

### Detection of signals of positive selection

To characterize signals of positive selection specific of the Lithuanian population we merged our original SNP genotyping data to that downloaded from the 1000 Genomes Project Phase3 dataset^16^ generating a pooled dataset of 264,950 autosomal SNPs distributed genome-wide in a total of 2,928 individuals. In particular, we included 20 populations from the 4 main geographical regions as described in the 1000 Genomes Project Phase3 dataset^16^: Africa including the Yoruba in Ibadan, Nigeria (YRI), Luhya in Webuye, Kenya (LWK), Gambian in Western Divisions in the Gambia (GWD), Mende in Sierra Leone (MSL), and Esan in Nigeria (ESN) populations; Europe including Utah residents with ancestry from northern and western Europe (CEU), Toscani in Italy (TSI), Finnish in Finland (FIN), British in England and Scotland (GBR), and Lithuanians (LT); East Asia including Han Chinese in Bejing, China (CHB), Japanese in Tokyo, Japan (JPT), Southern Han Chinese, China (CHS), Chinese Dai in Xishuangbanna, China (CDX), and Kinh in Ho Chi Minh City, Vietnam (KHV); South Asia including Gujarati Indians in Houston, Texas (GIH), Punjabi from Lahore, Pakistan (PJL), Bengali from Bangladesh (BEB), Sri Lankan Tamil from the UK (STU) and Indian Telugu from the UK (ITU). Genotyping data was subsequently phased with SHAPEIT2^37^.

Signatures of recent or ongoing positive selection were next investigated using the locus fixation index (*F*_*ST*_)^38^ and the XP-EHH statistic^39^ which were computed between all possible pairs of populations among LT, CEU, FIN, and YRI. *F*_*ST*_ values were calculated with vcftools v.0.1.13^40^. Negative *F*_*ST*_ values were converted to zero. XP-EHH was run using selscan v1.2.0a^41^. For each comparison a XP-EHH score per SNP was obtained and XP-EHH scores >2 were considered as indicative of positive selection. We selected as candidates for positive selection any genomic region with two or more SNPs located at the 0.1% top extreme of the XP-EHH genome-wide empirical distribution and with at least one SNP presenting an *F*_*ST*_ p-value < 0.01.

Older signals of selection were investigated through the Tajima´s D statistic, a widely used neutrality test that uses the site frequency spectrum^42^ and which have been suggested to capture selective sweeps occurring up to ~250,000 years ago^43^. The statistic was calculated with the PopGenome package implemented in R^44^ considering 100 kb sliding windows across all autosomal regions with a step size of 10 kb. Windows containing missing variants were ignored. As was done for *F*_*ST*_, extreme negative Tajima’s D values were identified considering the rank of the score in the genomic distribution. In particular, windows were sorted in ascending order based on the Tajima’s D values and we considered for further analysis those with empirical p-values less than 0.01.

Finally, gene and variant annotation in candidate regions for selection was performed with ANNOVAR^45^ using GRCh37 (hg19), RefSeqGene, dbSNP147^46^ and CADD version 1.3^47^.

### Ethics approval and consent to participate

This research was approved by the Vilnius Regional Research Ethics Committee No. 158200-05-329-79, date: 2011-05-03. All the study participants provided written informed consent.

## Supplementary information

Supplementary Material

## Data Availability

All the genotyping data generated in this study have been deposited at https://figshare.com/articles/Patterns_of_genetic_structure_and_adaptive_positive_selection_in_the_Lithuanian_population_from_high-density_SNP_data/7964159.
