# Supplementary material for: Patterns of genetic structure and adaptive positive selection in the Lithuanian population from high-density SNP data
Source: Sci Rep. 2019 Jun 24;9:9163. doi: 10.1038/s41598-019-45746-3 (PMC6591479; doi:10.1038/s41598-019-45746-3)
Supplement: Supplementary file 1 — Supplementary Material [file 41598_2019_45746_MOESM1_ESM.pdf]

Supplementary Material

Patterns of genetic structure and adaptive positive selection in the Lithuanian population from high- density SNP data

[1]A. Urnikyte, [2]A Flores-Bello, [3]M. Mondal, [1]A. Molyte, [2]D. Comas, [2]F. Calafell, [2*]E. Bosch, [1*]V. Kučinskas

[1]Department of Human and Medical Genetics, Biomedical Science Institute, Faculty of Medicine, Vilnius University, Santariskiu Street 2, LT-08661 Vilnius, Lithuania
[2]Institut de Biologia Evolutiva (UPF-CSIC), Departament de Ciències Experimentals i de la Salut, Universitat Pompeu Fabra, Parc de Recerca Biomèdica de Barcelona, Doctor Aiguader 88, 08003 Barcelona, Catalonia, Spain
[3] Estonian Biocentre, Tartu 51010, Estonia

* Corresponding authors

Supplementary Tables

Table S1. Lithuanian individuals filtered out in our study after successful genotyping.

Number	ID	Kinship	F	PCA outlier
1	356	with ID1401 (0.5)	-	yes
2	1401	with ID 356 (0.5)	-	yes
3	333	with ID 813 (0.5)	-	yes
4	813	with ID 333 (0.5)	-	yes
5	1158	with ID 1158 (0.5)	-	yes
6	1158	duplicate	-	yes
7	429	with ID 423 (0.2704)	-	no
8	423	with ID 429 (0.2704)	-	no
9	793	with ID 294 (0.2503)	-	no
10	294	with ID 793 (0.2503)	-	no
11	665	with ID 614 (0.2492)	-	no
12	614	with ID 665 (0.2492)	-	no
13	616	with ID 665 (0.2482)	-	no
14	348	with ID 376 (0.2476)	-	no
15	788	with ID 294 (0.2466)	-	no
16	1061	with ID 1056 (0.1403)	-	no
17	1056	with ID 1061 (0.1403)	-	no
18	197	with ID 246 (0.1295)	-	no
19	246	with ID 197 (0.1295)	-	no
20	1074	-	-	yes
21	1105	-	-	yes
22	972	-	0.02504	no
23	1271	-	0.01846	no
24	875	-	0.01573	no
25	781	-	0.01551	no

Supplementary Figures

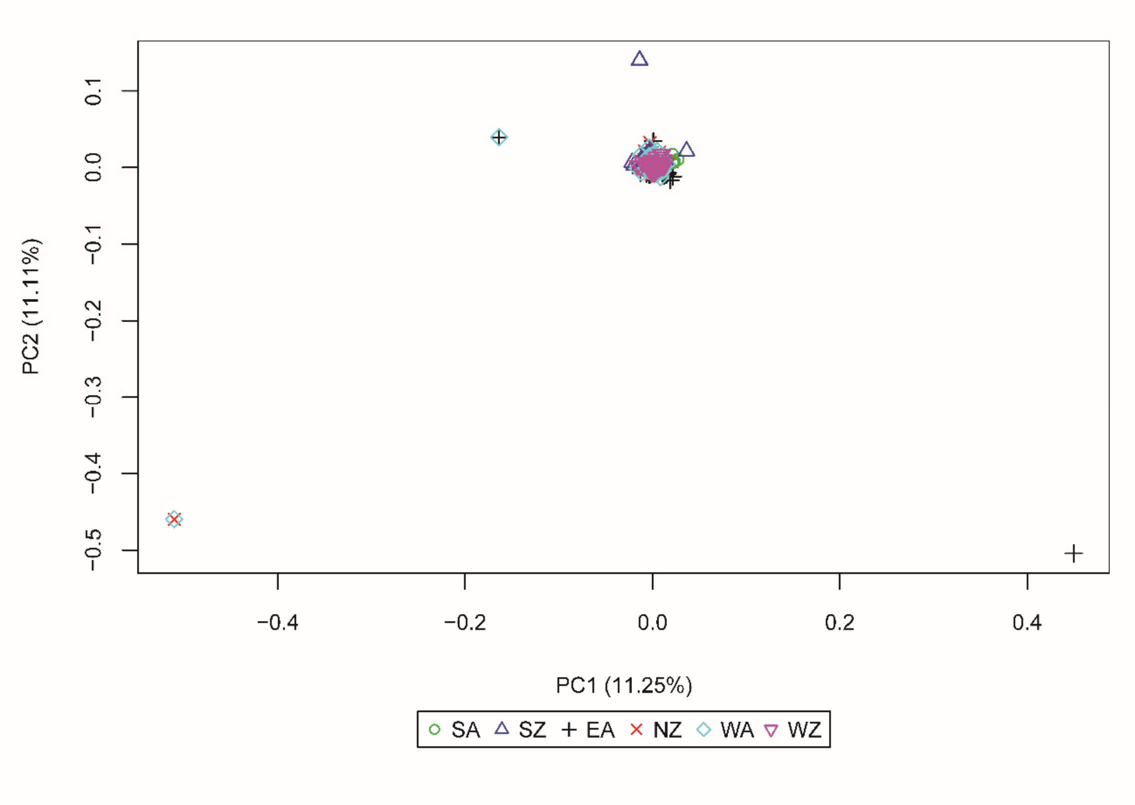

Figure S1. Principal component analysis (PCA) of the six ethnolinguistic groups of the Lithuanian population. Initial dataset with all samples successfully genotyped included. Principal components 1 and 2 are shown. NZ, North Zemaitija; SZ, South Zemaitija; WZ, West Zemaitija; EA, East Aukstaitija; SA, South Aukstaitija; WA, West Aukstaitija.

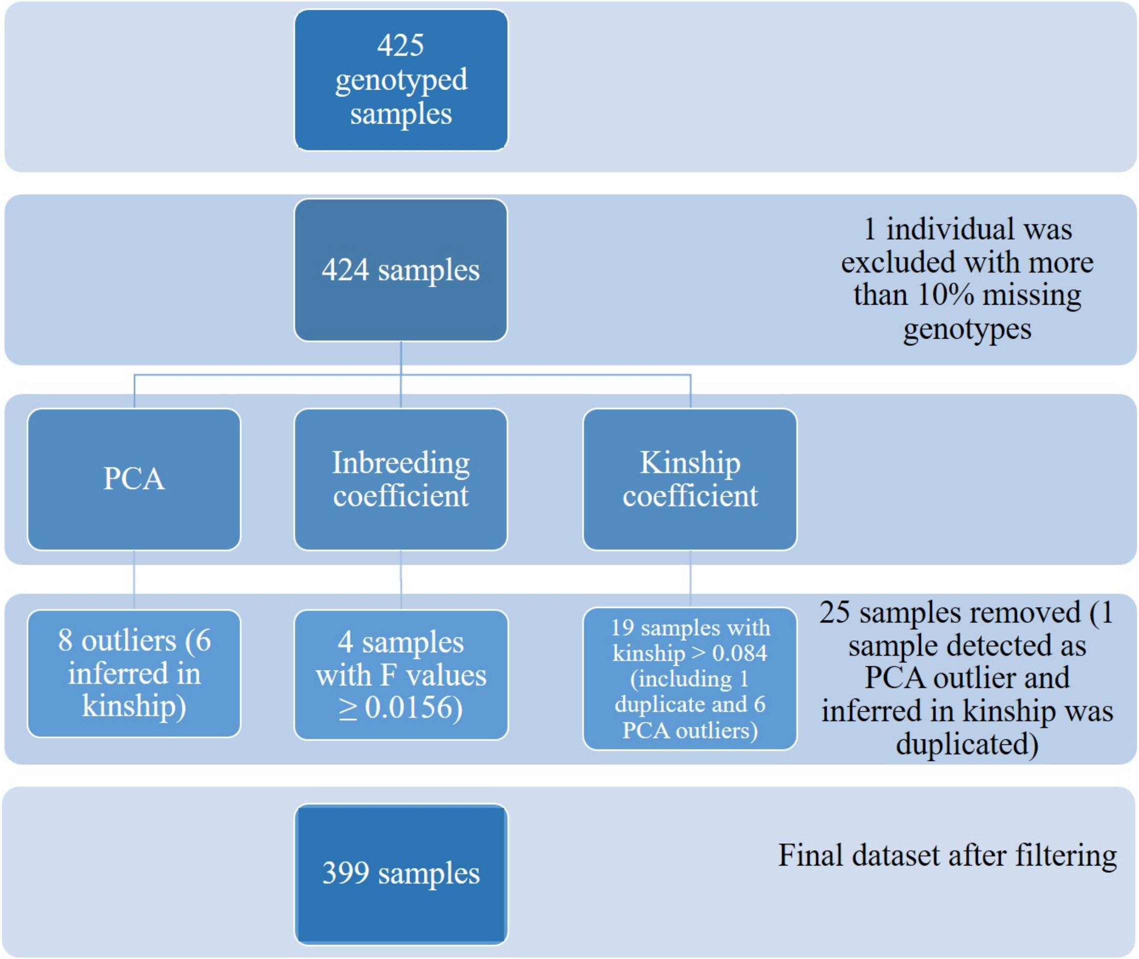

Figure S2. Flow chart with all filtering steps applied to obtain the final dataset of 399 Lithuanian samples.

a)

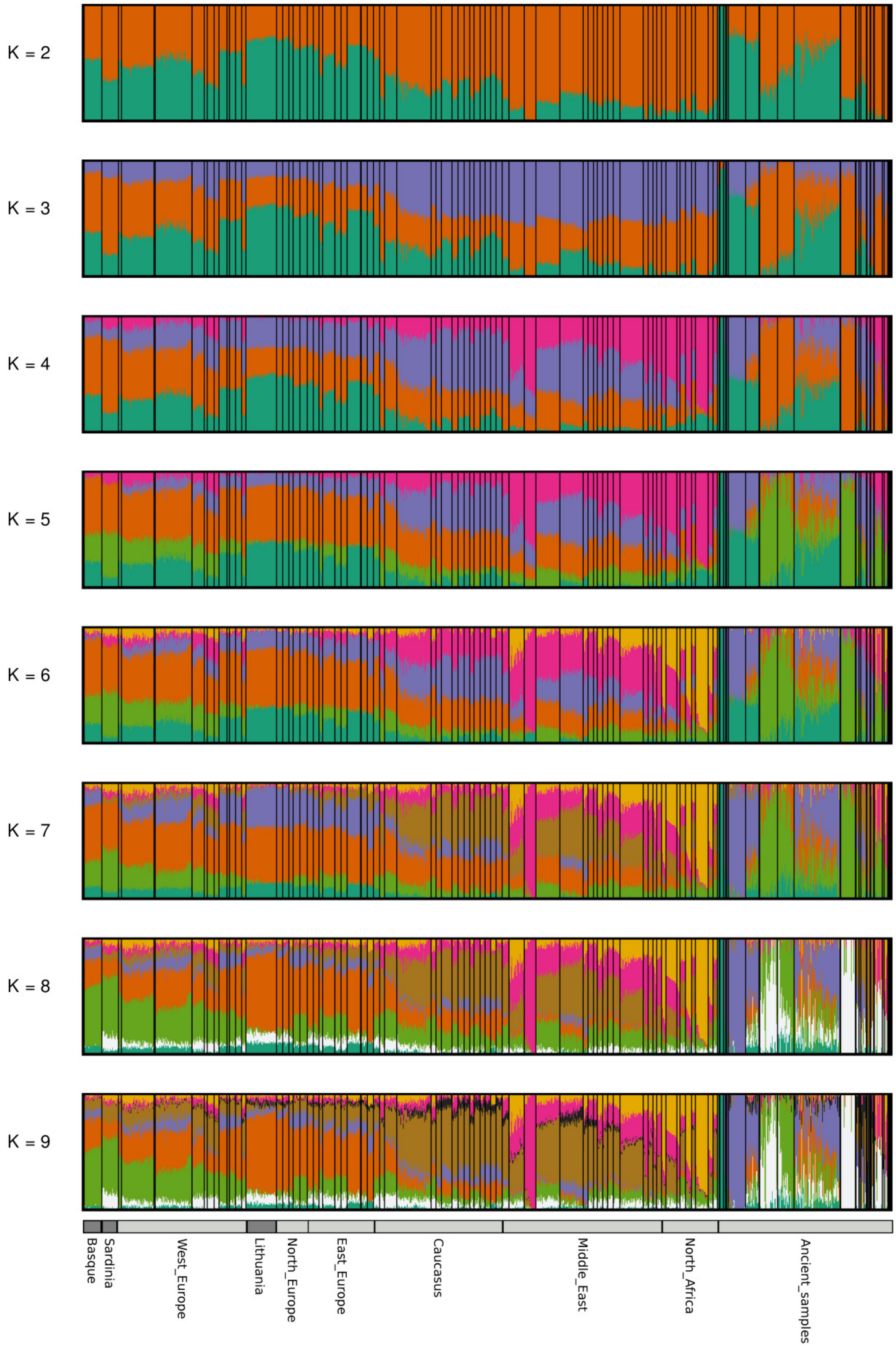
K = 2
K = 3
K = 4
K = 5
K = 6
K = 7
K = 8
K = 9
Basque
Sardinia
West_Europe
Lithuania
North_Europe
East_Europe
Caucasus
Middle_East
North_Africa
Ancient_samples

b)

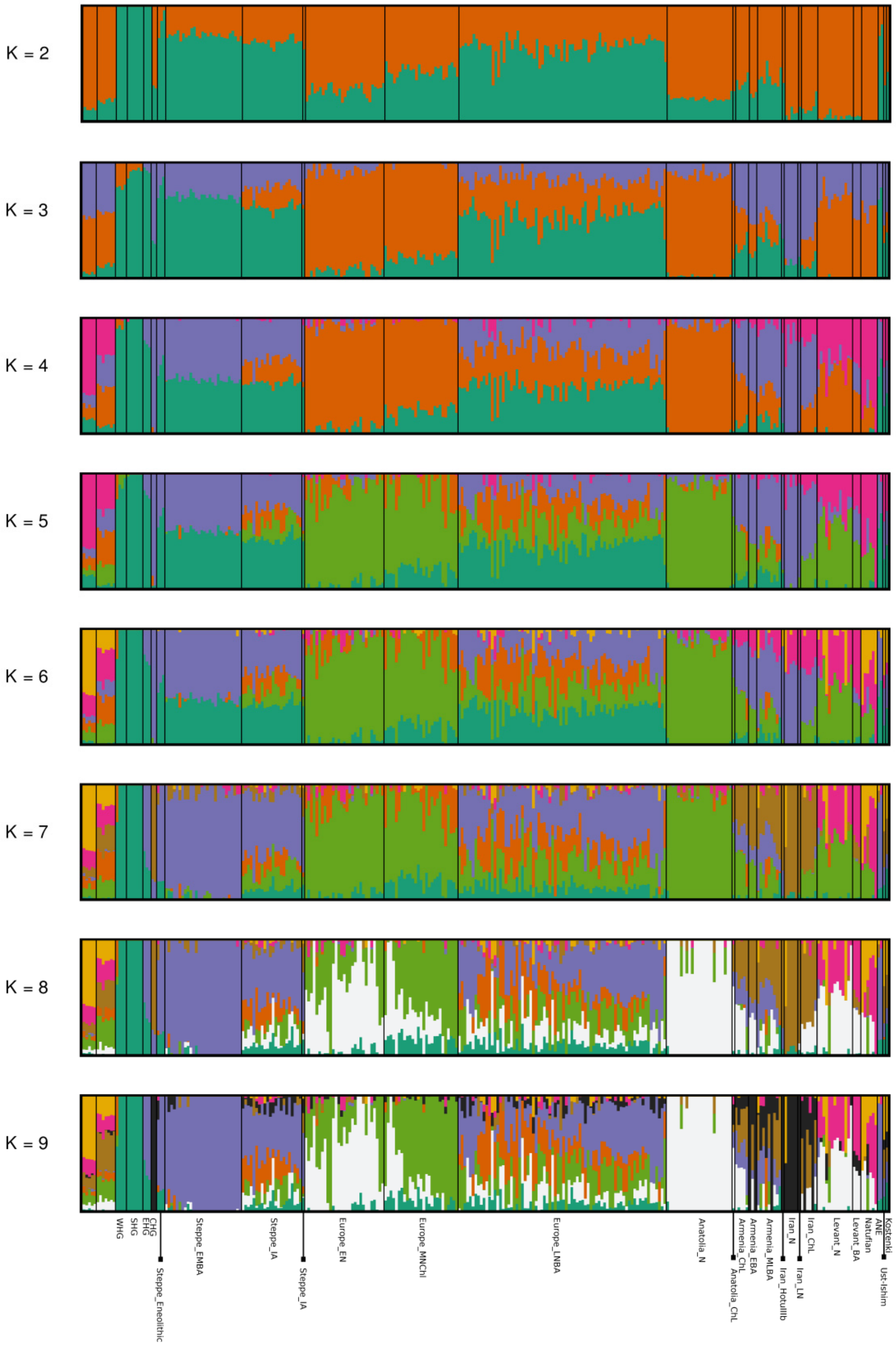

Figure S3. Admixture analysis. a) Admixture results from K=2 to K=9 including all present-day and ancient samples from the dataset. The lowest cross-validation error in the analysis was K = 4. Ancient samples are shown on the left. b) Zoom for ancient samples only.

Figure S4. Outgroup f3-statistics. Outgroup f3-statistics plot for each ancient group of interest in the form f3 (Mbuti; Ancient, X), being X a modern population. Values are represented in the x-axis for each modern population plotted in the y-axis. Only the top 60 populations with the highest values of f3 are shown. Lithuanian samples from this study are plotted as LT, whereas those from Lazaridis dataset[6] as Lithuanian.

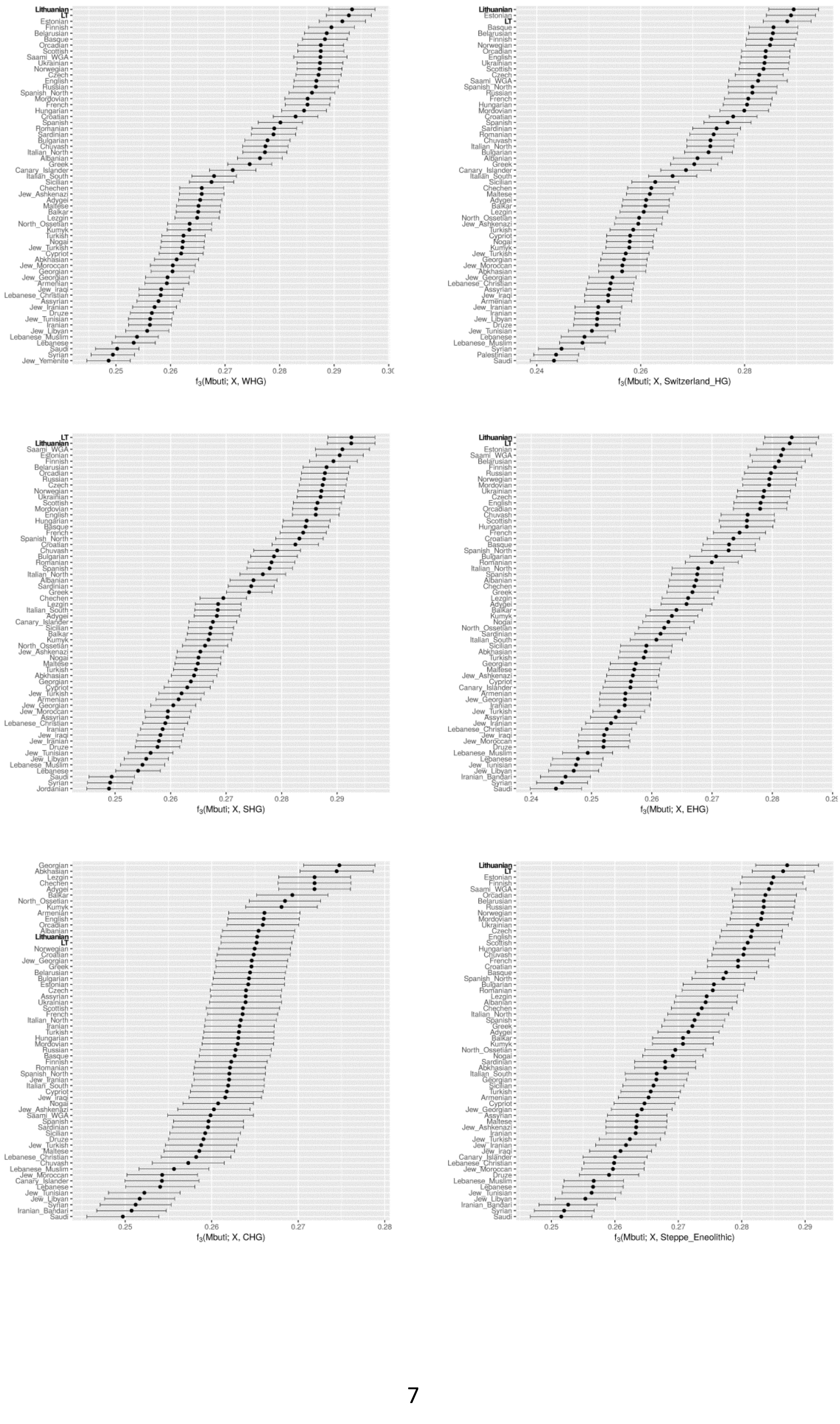

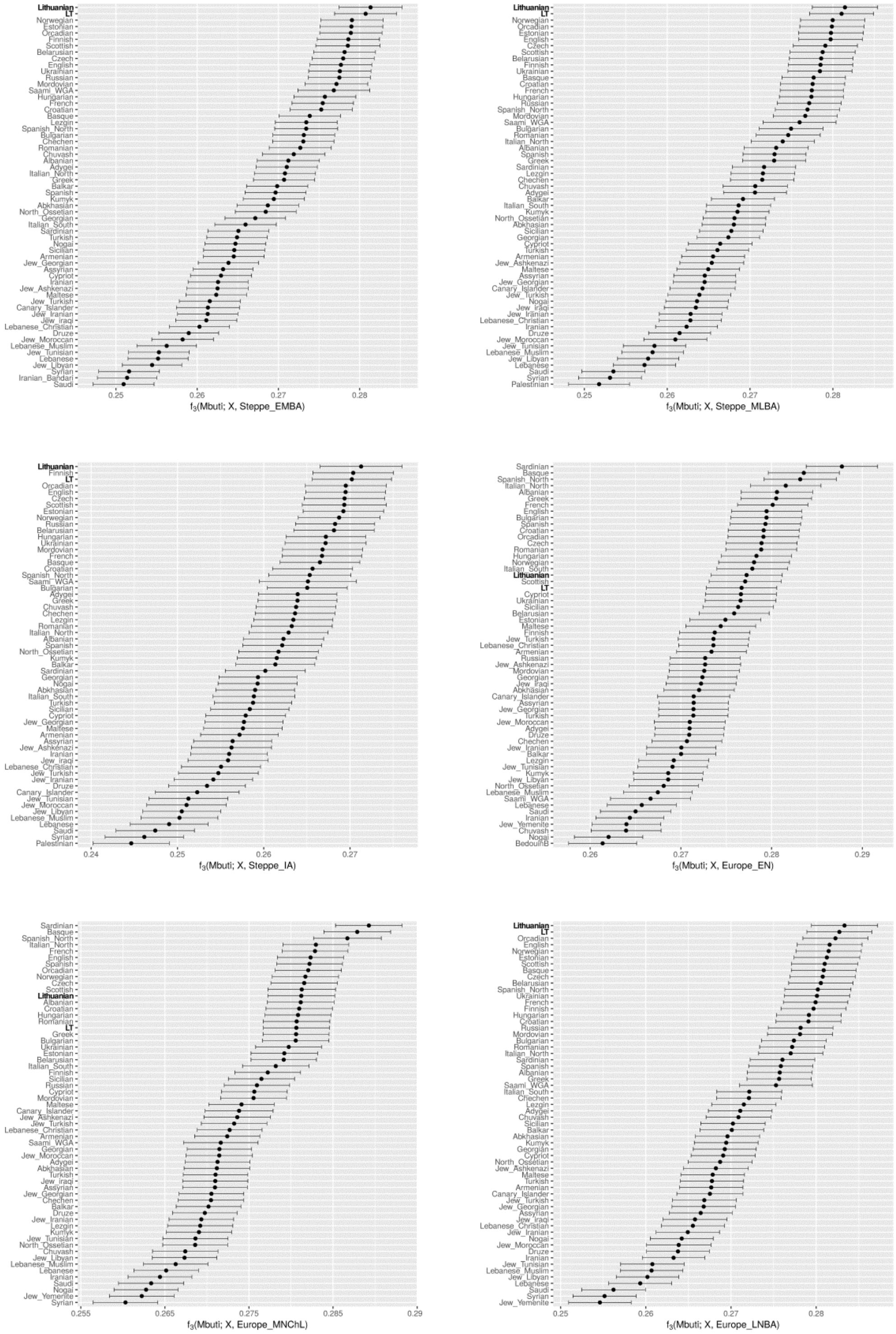

f3(Mbuti; X, Steppe_EMBA)
f3(Mbuti; X, Steppe_MLBA)
f3(Mbuti; X, Steppe_IA)
f3(Mbuti; X, Europe_EN)
f3(Mbuti; X, Europe_MNChL)
f3(Mbuti; X, Europe_LNBA)

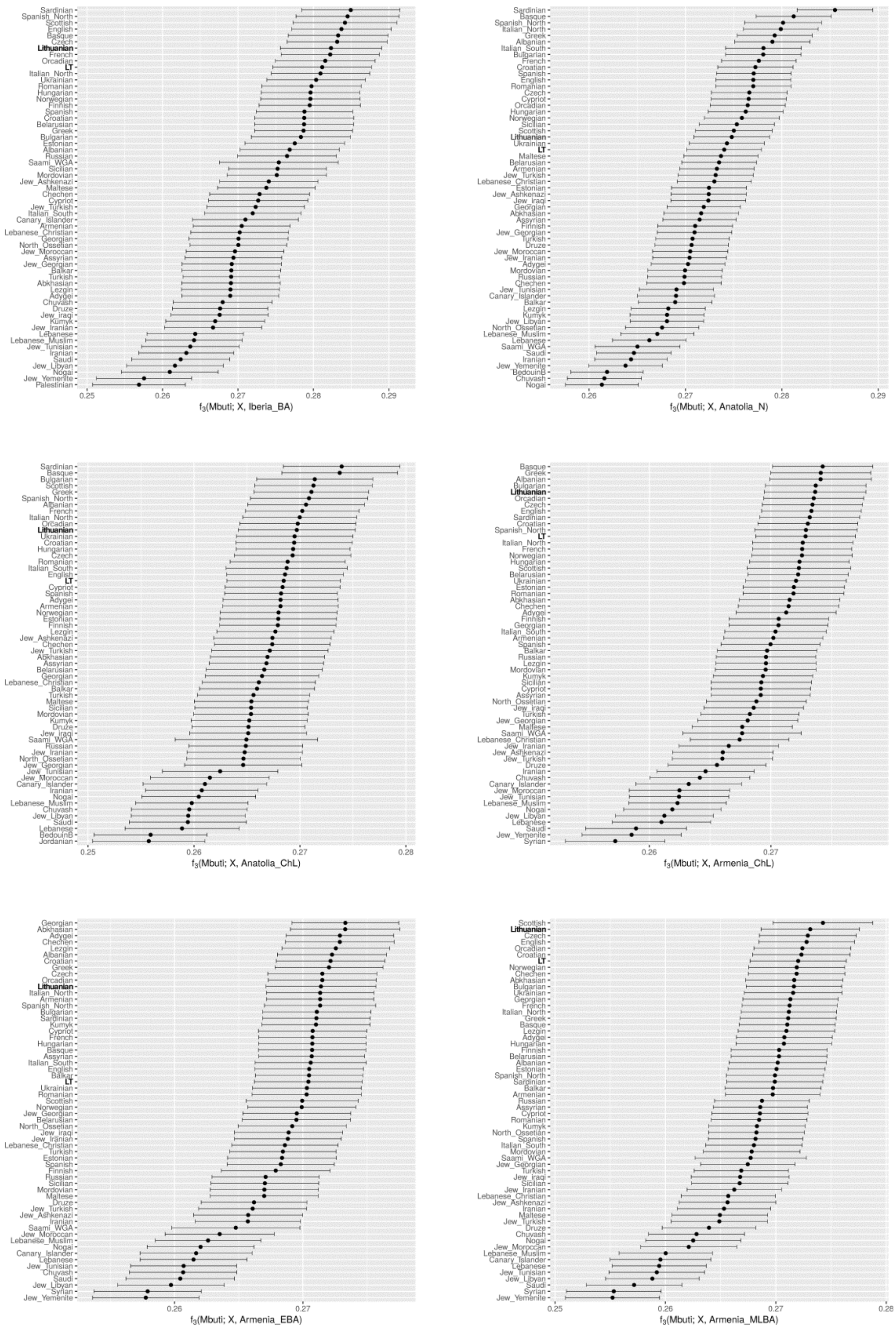
f3(Mbuti; X, Iberia_BA)
0.25 0.26 0.27 0.28 0.29
f3(Mbuti; X, Anatolia_N)
0.26 0.27 0.28 0.29
f3(Mbuti; X, Anatolia_ChL)
0.25 0.26 0.27 0.28
f3(Mbuti; X, Armenia_ChL)
0.26 0.27
f3(Mbuti; X, Armenia_EBA)
0.26 0.27
f3(Mbuti; X, Armenia_MLBA)
0.25 0.26 0.27 0.28

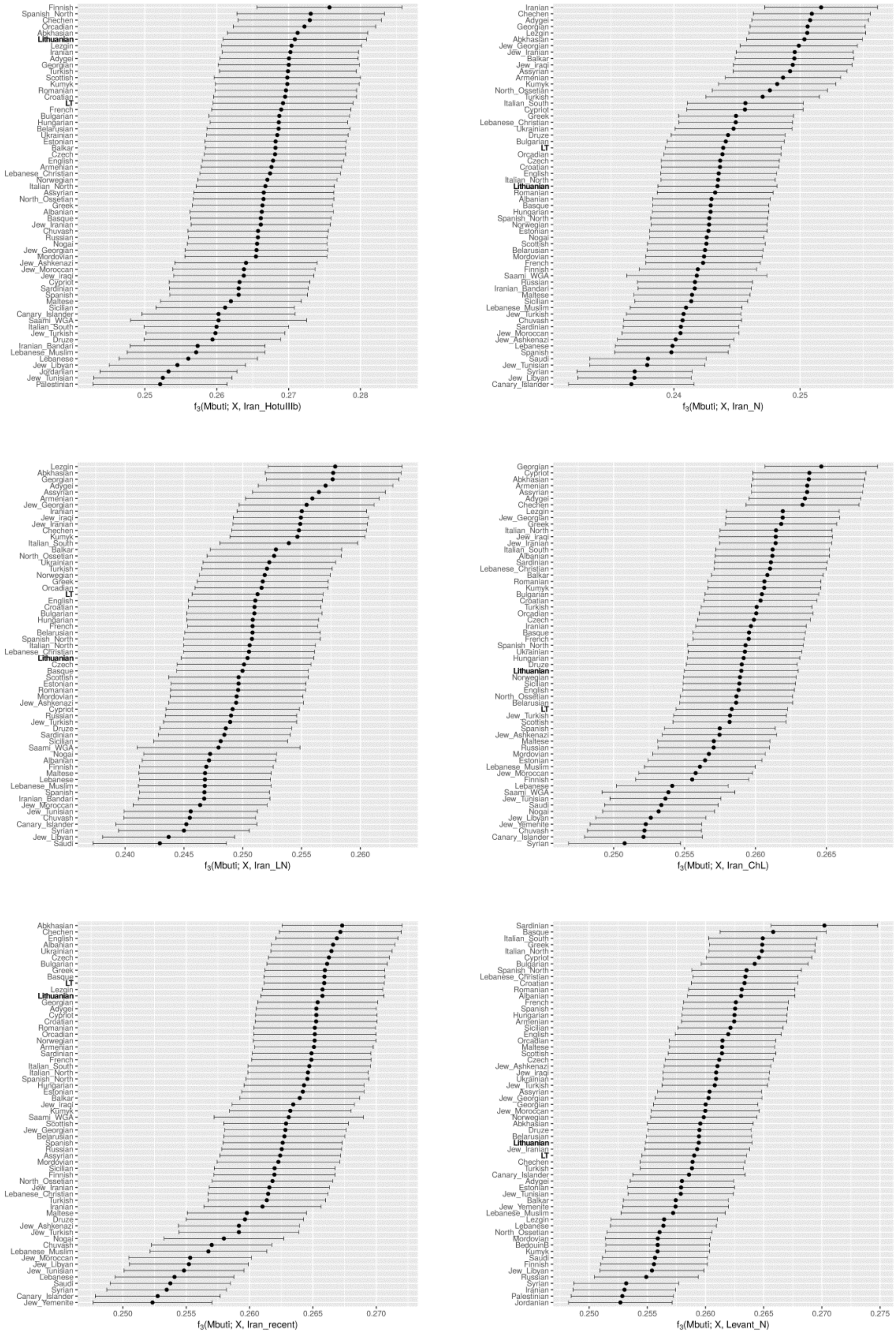
f3(Mbuti; X, Iran_HotuIIIb)
f3(Mbuti; X, Iran_N)
f3(Mbuti; X, Iran_LN)
f3(Mbuti; X, Iran_ChL)
f3(Mbuti; X, Iran_recent)
f3(Mbuti; X, Levant_N)

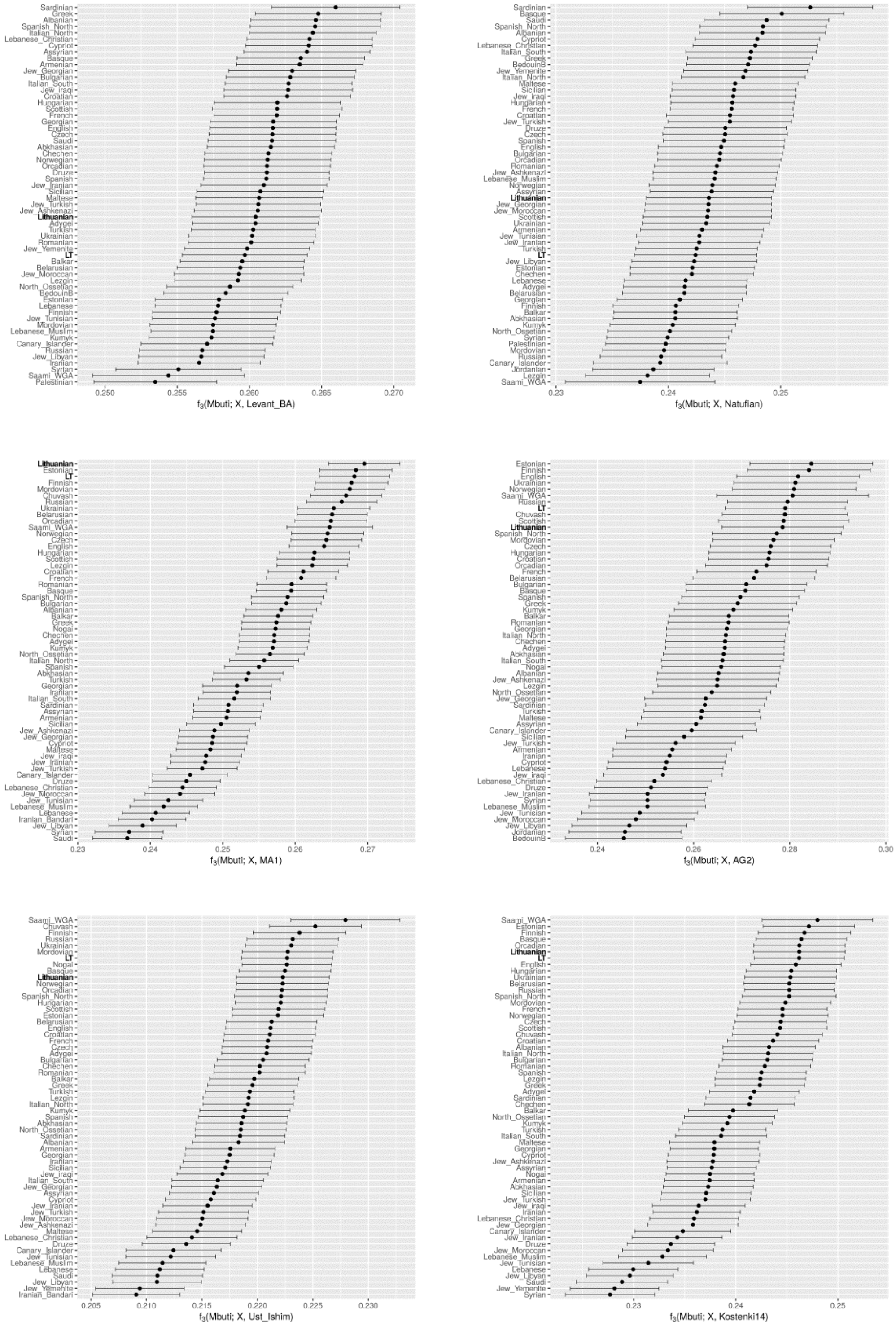
f3(Mbuti; X, Levant_BA)
f3(Mbuti; X, Natufian)
f3(Mbuti; X, MA1)
f3(Mbuti; X, AG2)
f3(Mbuti; X, Ust_Ishim)
f3(Mbuti; X, Kostenki14)

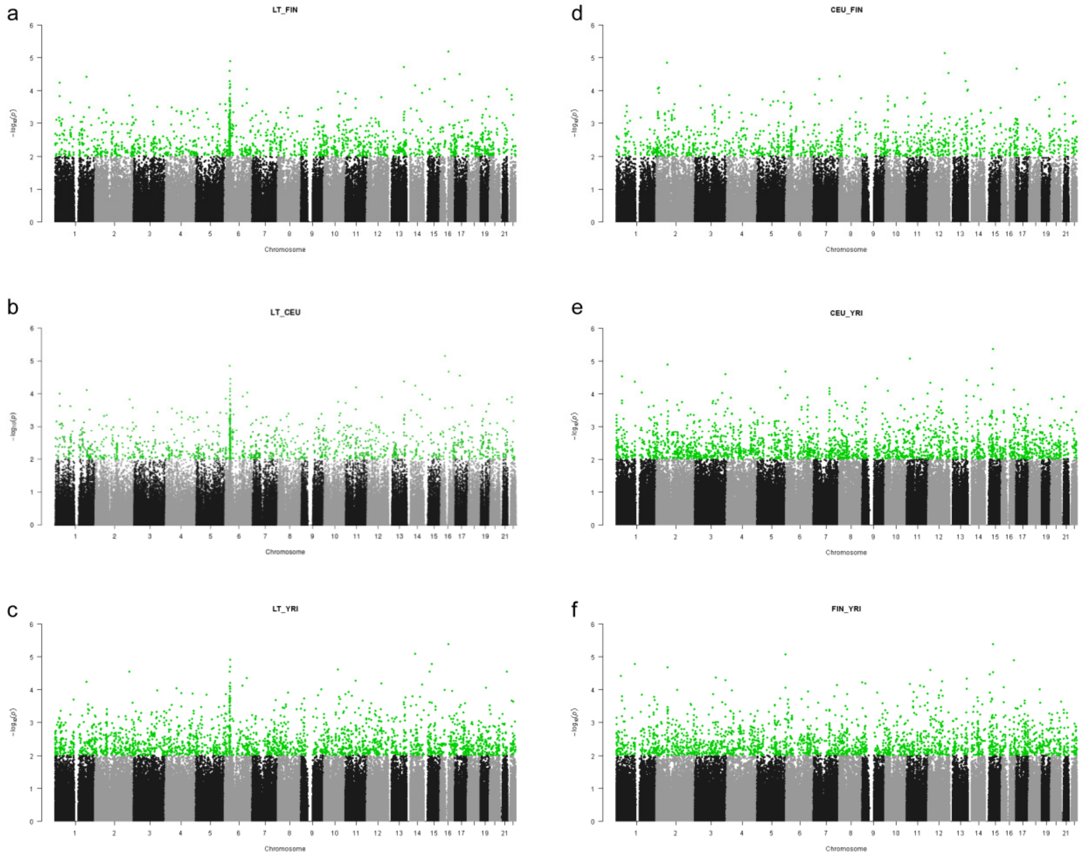

Figure S5. Manhattan plots of negative log transformed p-values for F_{ST} (a) F_{ST} between LT-CEU, (b) F_{ST} between LT-FIN, (c) F_{ST} between LT-YRI, (d) F_{ST} between CEU-FIN, (e) F_{ST} between CEU-YRI, (f) F_{ST} between FIN-YRI. In each plot, green dots indicate F_{ST} values with $p < 0.01$.

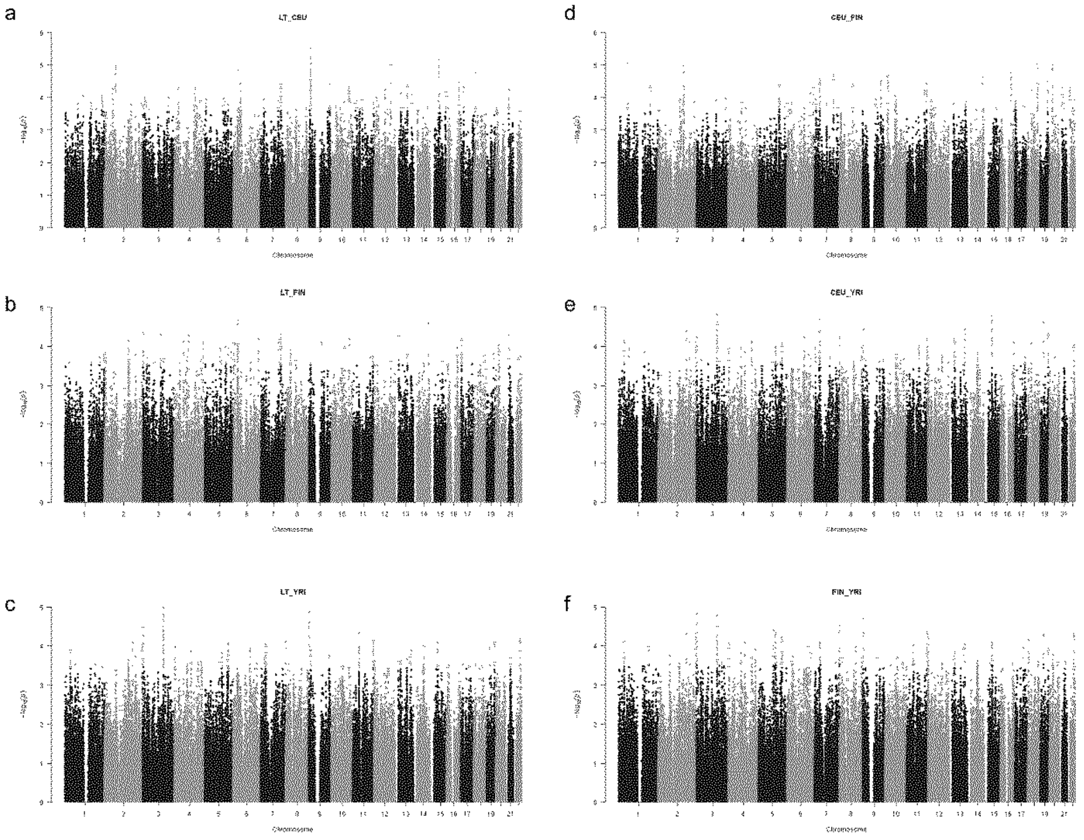

Figure S6. Manhattan plots of -log$_{10}$ transformed XP-EHH p-values across the autosomes. (a) XP-EHH in LT-CEU, (b) XP-EHH in LT-FIN, (c) XP-EHH in LT-YRI, (d) XP-EHH in CEU-FIN, (e) XP-EHH in CEU-YRI, (f) XP-EHH in FIN-YRI. In each plot, green dots indicate 0.1% outlier regions.

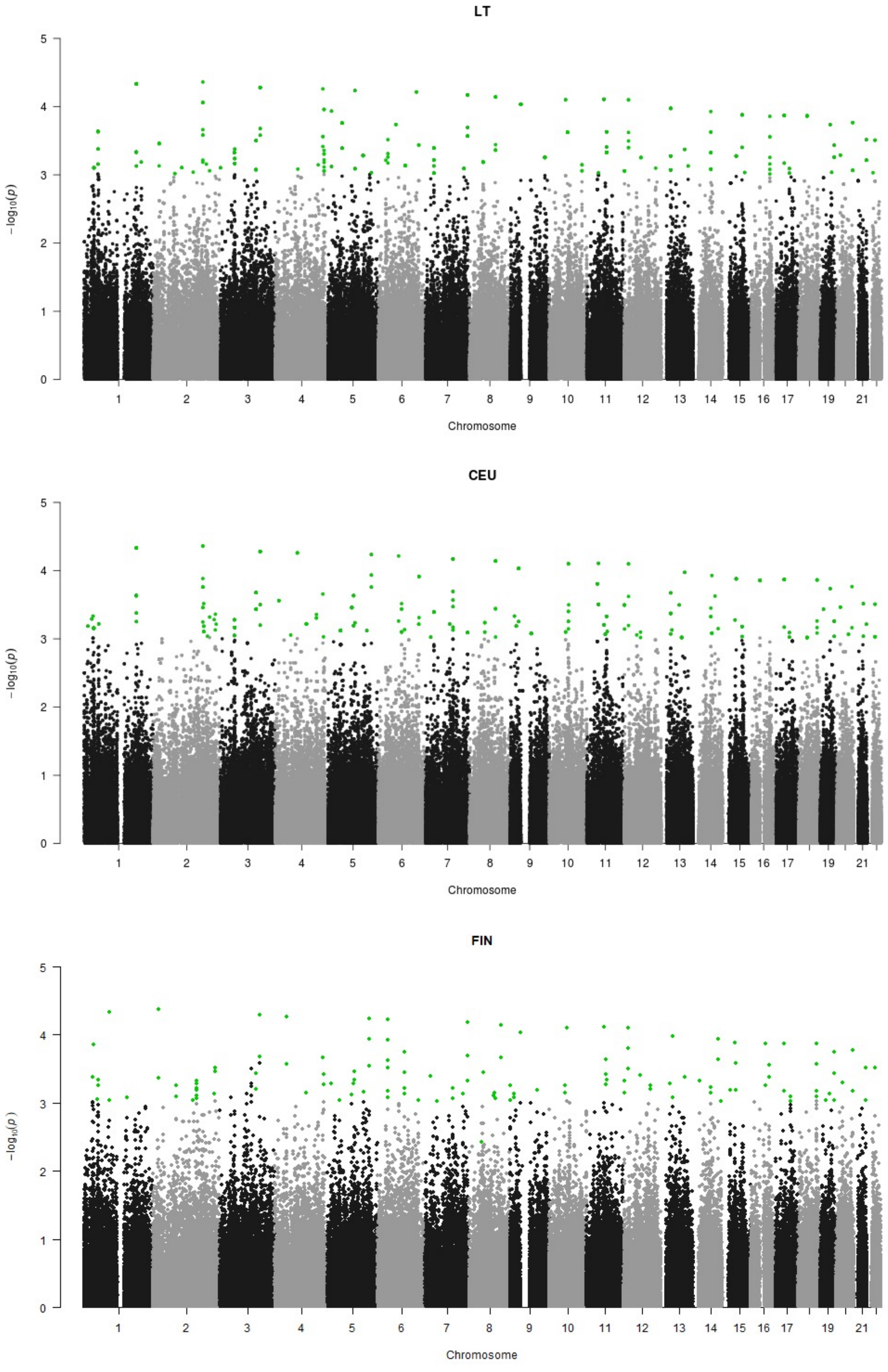
LT
CEU
FIN
$-\log_{10}(p)$
Chromosome
1 2 3 4 5 6 7 8 9 10 11 12 13 14 15 16 17 19 21

Figure S7. Manhattan plots of the log transformed Tajima's D p-values (a) In Lithuanians, (b) In CEU, (c) In FIN. In each plot, green dots indicate 0.1% outlier regions.

Supplementary Note 1

To characterize signals of positive selection specific of the Lithuanian population we merged our original SNP genotyping data to that downloaded from the 1000 Genomes Project Phase3 dataset[1] generating a pooled dataset of 264,950 autosomal SNPs distributed genome-wide in a total of 2,928 individuals from 20 populations and 4 main geographical regions: Africa including the Yoruba in Ibadan, Nigeria (YRI), Luhya in Webuye, Kenya (LWK), Gambian in Western Divisions in the Gambia (GWD), Mende in Sierra Leone (MSL), and Esan in Nigeria (ESN) populations; Europe including Utah residents with ancestry from northern and western Europe (CEU), Toscani in Italy (TSI), Finnish in Finland (FIN), British in England and Scotland (GBR), and Lithuanians (LT); East Asia including Han Chinese in Bejing, China (CHB), Japanese in Tokyo, Japan (JPT), Southern Han Chinese, China (CHS), Chinese Dai in Xishuangbanna, China (CDX), and Kinh in Ho Chi Minh City, Vietnam (KHV); South Asia including Gujarati Indians in Houston, Texas (GIH), Punjabi from Lahore, Pakistan (PJL), Bengali from Bangladesh (BEB), Sri Lankan Tamil from the UK (STU) and Indian Telugu from the UK (ITU).

Prior to the analysis of signatures of selection, we performed PCA and admixture analyses on the combined Lithuanian – 1000 Genomes Project Phase3 dataset (excluding those populations from the American continent) to verify the correct merging between datasets and to confirm the European context of the genetic diversity of the Lithuanian population. Principal component analysis (PCA) was carried out with independent pruned SNPs using SmartPCA from EIGENSOFT 7.2.1[2]. SNPs in linkage disequilibrium were removed with the indep-pairwise option of PLINK v1.07 using a window size of 50 SNPs, a step size of 5, and a r^2 threshold of 0.5[3].

When using the combined Lithuanian – 1000 Genomes Project Phase3 dataset, the first two PCs explained 52.7 % and 32.3 % of the variance, respectively, and showed a clear clustering of all populations according their continental origin. As expected, the Lithuanian population appeared within the European cluster (Figure SN1).

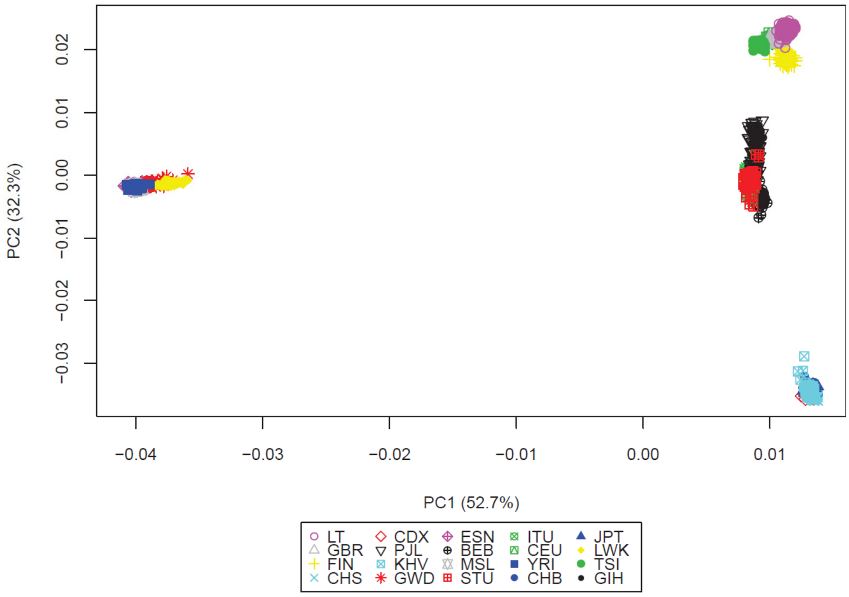

Figure SN1. Principal component analysis (PCA) of individuals from Lithuania and 19 external populations from the 1000 Genomes Project Phase3 dataset[1]. Principal components 1 and 2 are shown. Abbreviations as indicated the text.

Next, we just considered the six Lithuanian ethnolinguistic groups together with four European populations (CEU, FIN, GBR and TSI) from the 1000 Genomes project Phase3 dataset[1] and performed a new PCA using 158,633 SNPs (Figure SN2). The first PC, explaining 27.11% of the genetic variance, separated Lithuanians from all four European populations included, whereas the second PC, explaining 13.42 % of the genetic variance, separated the FIN population, more widely dispersed in the plot, from the remaining populations. To test any possible sample size bias, the PCA analysis was repeated twice with 100 randomly selected Lithuanian samples. All previously described patterns were maintained indicating no major effect due to the higher sample size of the Lithuanians when compared to the remaining European populations (results not shown).

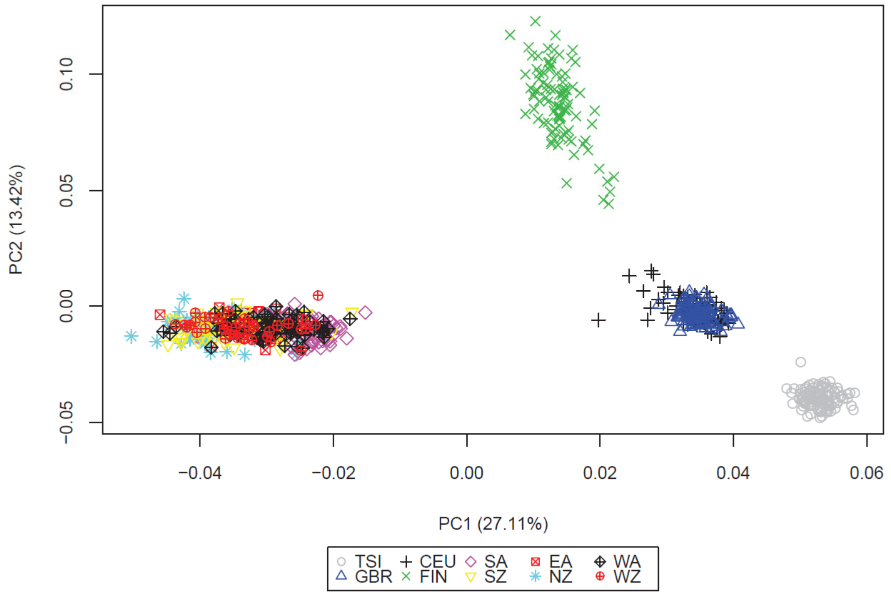

Figure SN2. Principal component analysis (PCA) of six ethnolinguistic groups in Lithuania and neighbouring European populations. The first two PCs are shown. EA – East Aukstaitija, SA - South Aukstaitija, WA – West Aukstaitija, NZ – North Zemaitija, SZ – South Zemaitija (SZ), WZ –West Zemaitija. CEU - Utah residents with ancestry from northern and western Europe, FIN - Finnish in Finland, GBR - British in England and Scotland and TSI – Toscani in Italy.

Subsequently, ancestry analysis was performed with ADMIXTURE v.1.3.0 varying the number of ancestral populations (*K*) between 2 and 9[4]. The best *K* was identified using the cross-error estimation implemented in ADMIXTURE. A total of 2,317 individuals from 20 populations and 187,447 SNPs were used in this global ADMIXTURE analysis (Figure SN3). The lowest cross-validation error was achieved with eight ancestry components. At K = 2, all African populations (yellow) were distinguished from populations in East Asia, Europe and South Asia (brown). At K = 3, a new ancestry component (green) distinguished Europe and South Asia from East Asia (brown) and Africa (yellow). At K = 4, a new component (yellow) distinguished all the European populations (including LT) from South Asians and it is not till K = 6 that an ancestry component appears specifically at high proportion in the Lithuanian population (fuchsia). At K = 8 (the lowest cross-validation error), Lithuanians were characterized by a predominant ancestral genetic component (green), which was present at similar proportions across the six ethnolinguistic groups and shared at low proportions with

other neighbouring Europeans (CEU, GBR and FIN). Potential size biases were tested by subsampling 120 Lithuanian individuals twice, with no major effects (Figure SN4).

References

1 Auton, A. *et al.* A global reference for human genetic variation. *Nature* **526**, 68-74, doi:10.1038/nature15393 (2015).

2 Patterson, N., Price, A. L. & Reich, D. Population structure and eigenanalysis. *PLoS Genet* **2**, e190, doi:10.1371/journal.pgen.0020190 (2006).

3 Purcell, S. *et al.* PLINK: a tool set for whole-genome association and population-based linkage analyses. *Am J Hum Genet* **81**, 559-575, doi:10.1086/519795 (2007).

4 Alexander, D. H., Novembre, J. & Lange, K. Fast model-based estimation of ancestry in unrelated individuals. *Genome Res* **19**, 1655-1664, doi:10.1101/gr.094052.109 (2009).

Figure SN3. ADMIXTURE analysis of Lithuanians and 19 external populations from the 1000 Genomes Project Phase3 dataset[1]. ADMIXTURE plots from K = 2 to K = 9 are shown. Individuals are represented as vertical coloured bars, in which each different coloured segment represents the proportion of an individual's ancestry derived from one of the K populations.

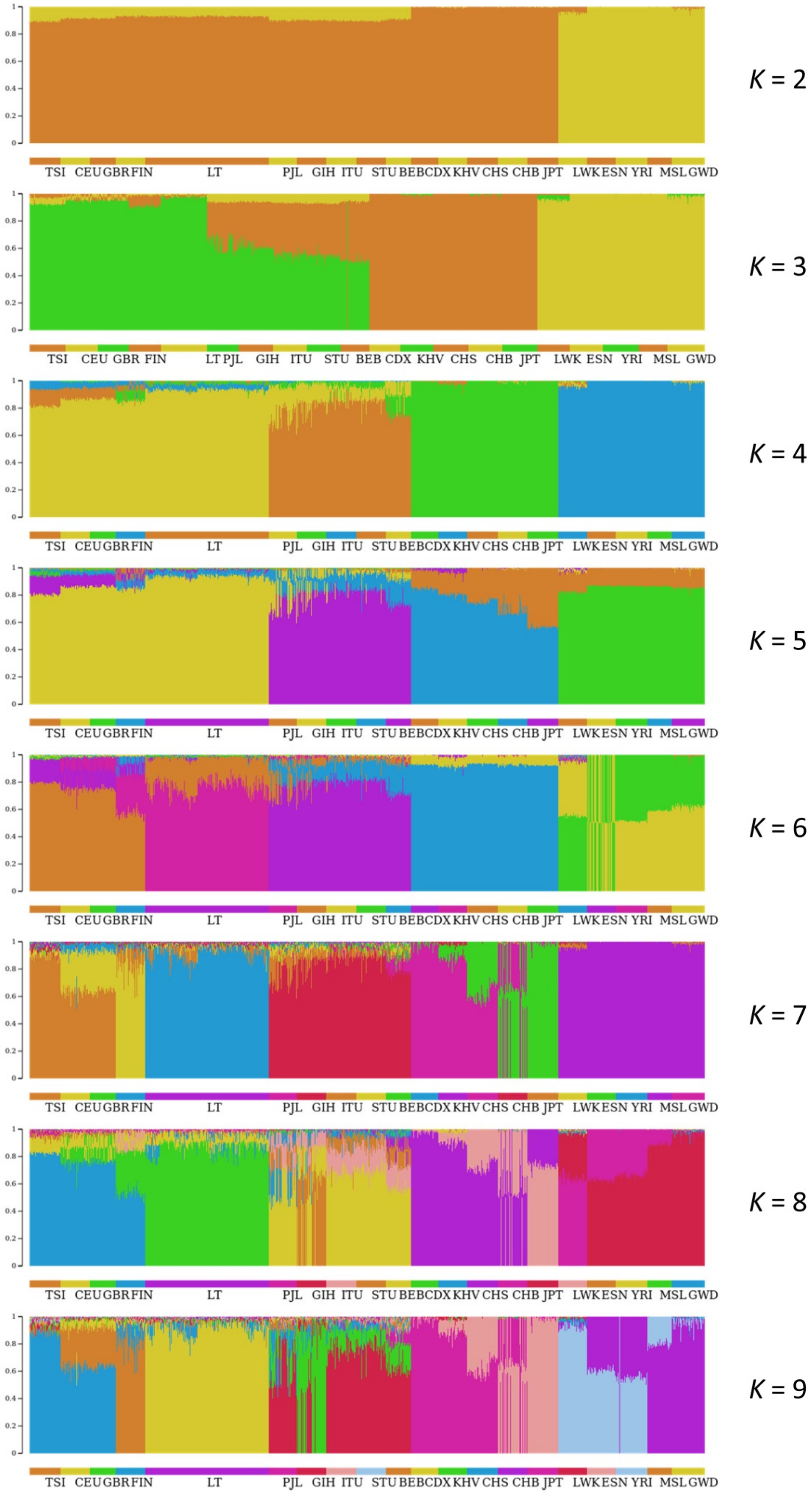
K = 2
TSI CEUGBRFIN LT PJL GIH ITU STU BEBCDXKHV CHS CHB JPT LWKESN YRI MSL GWD
K = 3
TSI CEU GBR FIN LT PJL GIH ITU STU BEB CDX KHV CHS CHB JPT LWK ESN YRI MSL GWD
K = 4
TSI CEUGBRFIN LT PJL GIH ITU STU BEBCDXKHV CHS CHB JPT LWKESN YRI MSL GWD
K = 5
TSI CEUGBRFIN LT PJL GIH ITU STU BEBCDXKHV CHS CHB JPT LWKESN YRI MSL GWD
K = 6
TSI CEUGBRFIN LT PJL GIH ITU STU BEBCDXKHV CHS CHB JPT LWKESN YRI MSL GWD
K = 7
TSI CEUGBRFIN LT PJL GIH ITU STU BEBCDXKHV CHS CHB JPT LWKESN YRI MSL GWD
K = 8
TSI CEUGBRFIN LT PJL GIH ITU STU BEBCDXKHV CHS CHB JPT LWKESN YRI MSL GWD
K = 9
TSI CEUGBRFIN LT PJL GIH ITU STU BEBCDXKHV CHS CHB JPT LWKESN YRI MSL GWD

Figure SN4. ADMIXTURE plots from K = 2 to K = 9 after subsampling twice 120 LT individuals when using 19 external populations from the 1000 Genomes Project Phase3 dataset[1]. (a) Subsample 1; (b) Subsample 2. The lowest cross-validation error was obtained at K = 8 (CV error = 0.52275).

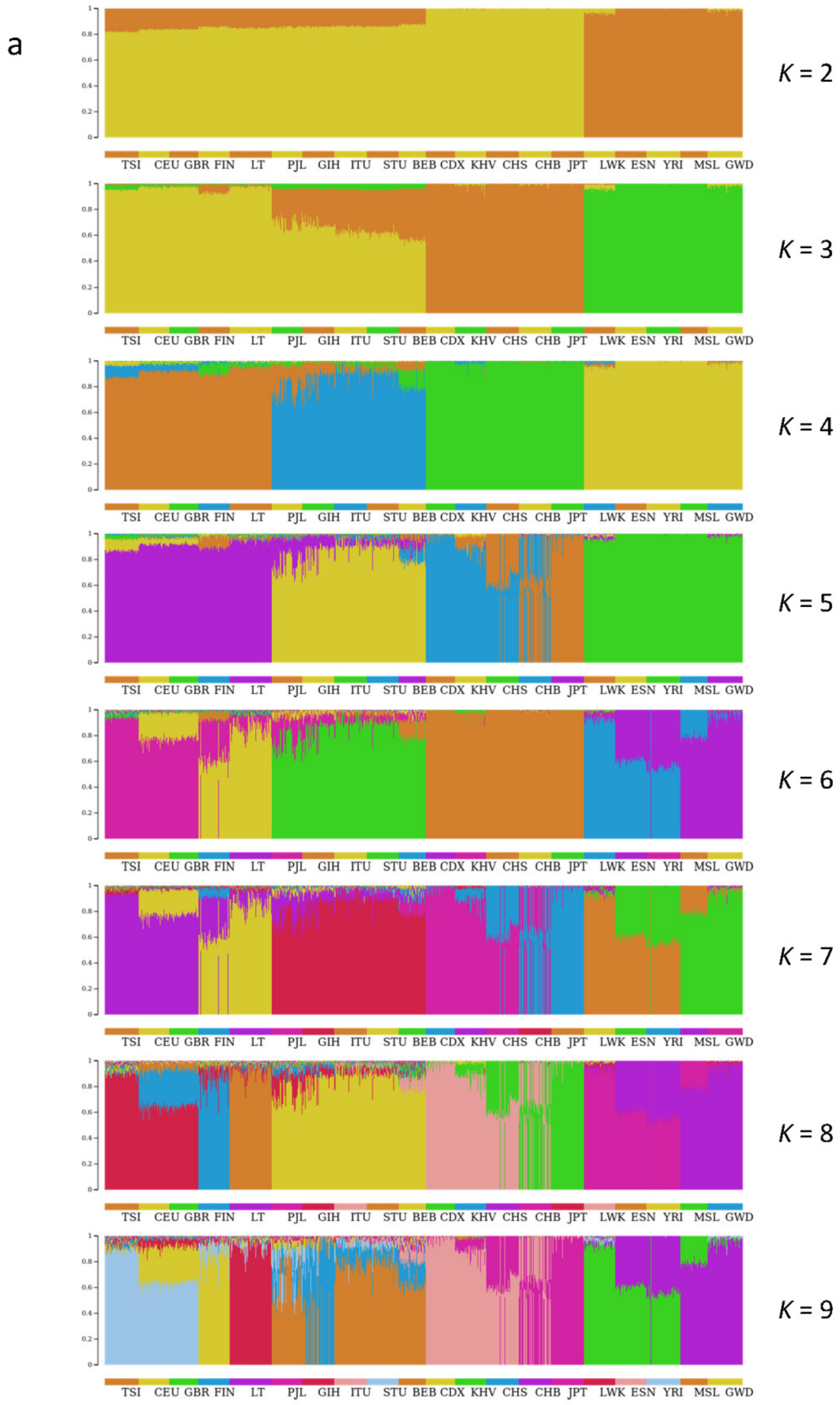

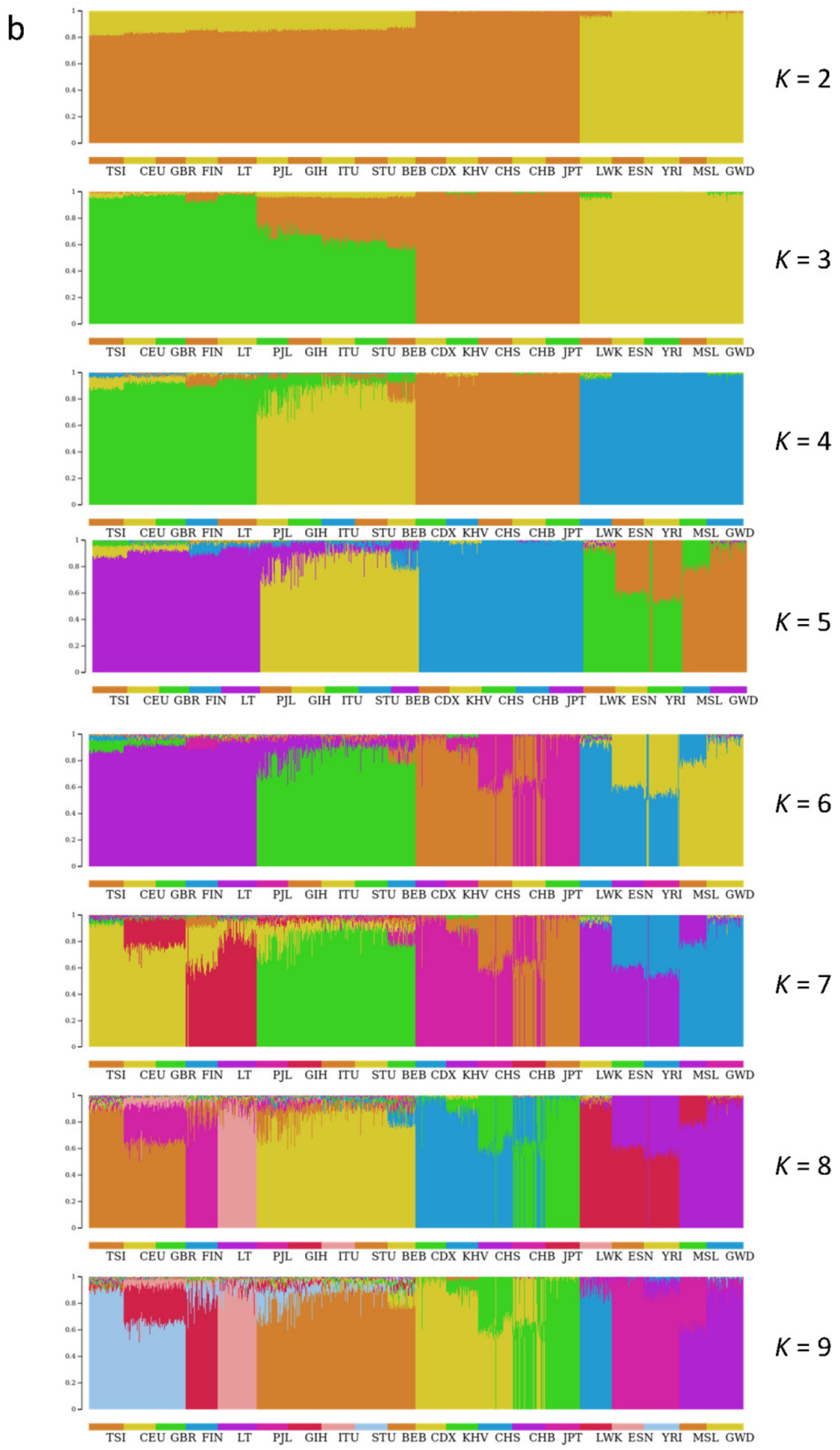
b
K = 2
K = 3
K = 4
K = 5
K = 6
K = 7
K = 8
K = 9
TSI CEU GBR FIN LT PJL GIH ITU STU BEB CDX KHV CHS CHB JPT LWK ESN YRI MSL GWD
